# TRIM26-Mediated Ubiquitination and Degradation of DDRGK1 Suppress NSCLC Progression by Inhibiting DNA Repair and Promoting Apoptosis

**DOI:** 10.3390/ijms272110014

**Published:** 2026-11-08

**Authors:** Yanmin Guo, Qifan Guo, Jingjin Yu, Yu Cao, Dexin Rao, Ziruo Guo, Binglong Li, Fanjian Jia, Qian Liang, Yang Zhuang, Shiwen Xu, Yaxiang Wang, Miao Wang, Yusheng Cong

**Affiliations:** Zhejiang Key Laboratory of Medical Epigenetics, Key Laboratory of Aging and Cancer Biology of Zhejiang Province, School of Basic Medical Sciences, Hangzhou Normal University, Hangzhou 311121, China

**Keywords:** NSCLC, DDRGK1, TRIM26, UFL1, ubiquitination, UFMylation

## Abstract

The high incidence and poor prognosis of non-small-cell lung cancer (NSCLC) underscore the urgent need to elucidate the molecular mechanisms underlying its progression and chemoresistance. Here, we investigated the role of DDRGK domain-containing protein 1 (DDRGK1) in NSCLC and identified a novel regulatory axis governing its protein stability. Clinically, DDRGK1 was markedly upregulated in NSCLC tissues, and elevated DDRGK1 expression correlated with advanced tumor stage, poor overall survival, and chemoresistance. Functionally, *DDRGK1* knockout significantly suppressed NSCLC cell growth, an effect reversed by DDRGK1 re-expression. Mechanistically, tripartite motif-containing protein 26 (TRIM26) was identified as the E3 ubiquitin ligase that catalyzed K48-linked polyubiquitination of DDRGK1 at K267, promoting its degradation. This modification antagonized E3 UFM1-protein ligase 1 (UFL1)-mediated UFMylation at the same residue, thereby fine-tuning DDRGK1 stability. Functional enrichment analysis showed that DDRGK1-interacting proteins and proteins differentially expressed in *DDRGK1*-knockout cells were predominantly associated with the DNA damage response and apoptosis. Consistently, *DDRGK1* knockout suppressed ATR phosphorylation and augmented cisplatin-induced apoptosis in NSCLC cells. Collectively, these findings define the regulatory mechanism of DDRGK1, establish it as a key regulator of NSCLC progression, and highlight its potential as a therapeutic target for overcoming chemoresistance.

## 1. Introduction

Lung cancer accounts for about one-fifth of global cancer deaths (GLOBOCAN 2024) [1], with non-small-cell lung cancer (NSCLC) comprising about 85% of cases. Nearly 70% of these cases, typically asymptomatic before onset, are diagnosed at locally advanced or metastatic stages [2]. Targeted therapy and immunotherapy have advanced NSCLC treatment [3], but targeted therapy depends on driver gene mutations, and immunotherapy alone has low response rates; most patients eventually develop resistance and require chemotherapy. Platinum-based chemotherapy plays an indispensable role in NSCLC therapy [4]. However, about 50% of patients with advanced NSCLC exhibit intrinsic resistance to cisplatin-based therapy [5]. Therefore, understanding the mechanisms of NSCLC chemoresistance is crucial for developing effective treatment strategies and improving patient prognosis [6].

Ubiquitin and ubiquitin-like modifiers are important post-translational protein modification that play essential roles in NSCLC progression [7,8,9,10]. By regulating substrate stability, conformation, localization, and activity, ubiquitination is involved in multiple oncogenic processes, including proliferation, migration, metabolic reprogramming, immune evasion, and chemoresistance [7,11]. Within this system, E3 ubiquitin ligases determine substrate specificity, which makes them key determinants of protein turnover [12]. TRIM26 is an RING-type E3 ubiquitin ligase of the TRIM family, characterized by a conserved N-terminal RBCC domain and a C-terminal PRYSPRY domain [13]. By ubiquitinating specific substrates, TRIM26 participates in various cellular processes, including tumorigenesis and immune regulation. In cancer, the role of TRIM26 depends largely on substrate identity. In colorectal cancer (CRC), TRIM26 suppresses CRC cell proliferation and colony formation by binding the C-terminus of p53 and accelerates its proteasomal degradation [14]. In osteosarcoma, TRIM26 acts as a tumor suppressor by destabilizing RACK1 and dampening MEK/ERK signaling [15]. These findings demonstrate the substrate-dependent and context-specific duality of TRIM26 in cancer. UFMylation is a newly identified ubiquitin-like modification that conjugates ubiquitin-fold modifier 1 (UFM1) to substrates through a dedicated cascade comprising E1 enzyme UBA5, E2 enzyme UFC1, and the sole E3 ligase UFL1 [16,17]. UFMylation is most prominently associated with endoplasmic reticulum (ER) homeostasis, ribosome-associated quality control, and the cell response to genotoxic stress [16,18,19,20]. DDRGK1 (DDRGK domain-containing protein 1, also known as C20orf116 or UFBP1) is an ER-resident adaptor. It anchors UFL1 to the ER membrane, and the resulting complex UFMylates substrates such as RPL26 and RPN1 [20]. Loss of DDRGK1 destabilizes IRE1α, shifts the UPR toward the PERK-eIF2α-CHOP apoptotic pathway, and sensitizes cells to ER stress-induced death [21]. DDRGK1 also supports p53 stabilization. UFL1-mediated UFMylation of p53 antagonizes MDM2-mediated ubiquitination, and *DDRGK1* depletion downregulates p53 [22]. Moreover, DDRGK1 and UFL1 also relocalize to nucleolar caps upon ribosomal DNA damage, where they are required for ATM activation and rDNA silencing [23]. Given these diverse functions, it is not surprising that dysregulation of UFMylation is increasingly implicated in cancer. In osteosarcoma, DDRGK1 is overexpressed and protects NRF2 from KEAP1-mediated ubiquitination, thereby restraining oxidative stress and weakening the response to doxorubicin and etoposide [24]. Recently, small molecules that disrupt the DDRGK1-UFL1 interface were shown to suppress glioblastoma growth and sensitize tumor cells to temozolomide and radiotherapy [25], suggesting that DDRGK1 may be a druggable node. However, the clinical relevance of DDRGK1 has not been established in NSCLC.

In this study, we explored the role and regulation mechanism of DDRGK1 in NSCLC. Elevated DDRGK1 correlated with advanced stage, poor survival, and chemoresistance. TRIM26 mediated K48-linked ubiquitination of DDRGK1 at K267, inducing its degradation and antagonizing UFL1-mediated UFMylation at the same residue. DDRGK1 supported ATR-mediated DNA repair; its knockout impaired ATR phosphorylation and enhanced cisplatin-induced apoptosis. These findings define a DDRGK1 regulatory mechanism and highlight DDRGK1 as a potential therapeutic target in NSCLC.

## 2. Results

### 2.1. DDRGK1 Protein Is Significantly Upregulated in Patients with NSCLC and Accelerates NSCLC Progression

To address the functional role of DDRGK1 in NSCLC, we first evaluated its expression using the Cancer Genome Atlas (TCGA) and Clinical Proteomic Tumor Analysis Consortium (CPTAC) datasets. Analysis of *DDRGK1* mRNA expression in lung adenocarcinoma (LUAD) and lung squamous cell carcinoma (LUSC) using the TCGA database showed no significant differences compared with normal lung tissues (Figure 1A). In contrast, analysis of its protein expression using the CPTAC database revealed that DDRGK1 protein was markedly upregulated in LUAD and LUSC specimens (Figure 1B). Of note, the *DDRGK1* mRNA levels in the CPTAC dataset also did not show a significant increase (Figure S1A). DDRGK1 is classified as a pan-essential gene in DepMap. Analysis of the DepMap database revealed that *DDRGK1* knockout inhibited the growth of NSCLC cell lines, which display negative Chronos scores (Figure S1B). We also compared DDRGK1 protein expression levels in NSCLC cells with different metastatic abilities. Compared to non-metastatic NSCLC cells (H1975, A549, and H23), metastatic NSCLC cells (H1299, H1944, H2126, and H838) showed a significant upregulation in DDRGK1 protein levels (Figure 1C). These data indicate that elevated DDRGK1 protein expression is implicated in the development and progression of NSCLC.

To assess DDRGK1 protein expression in human specimens, LUAD tissue microarray slides (Table S1) were used for immunofluorescence staining and analysis. The results show that the protein expression of DDRGK1 was much higher in LUAD cases than in normal tissues (Figure 1D,E). Most notably, this upregulation was observed at a higher frequency in late-stage than in early-stage LUAD tissues (Figure 1F). LUAD patients with metastases have shorter survival than their non-metastasis counterparts (HR = 3.172, 95% CI = 1.724–5.836) (Figure 1G). Correspondingly, DDRGK1 protein levels were higher in metastases tissues (Figure 1H). The Kaplan–Meier survival curves and log-rank test show that high DDRGK1 protein expression was significantly correlated with poorer overall survival (OS) in LUAD (HR = 2.208, 95% CI = 1.373–3.549) (Figure 1I). This correlation was also observed in patients who received chemotherapy, where high DDRGK1 expression similarly predicted inferior survival outcomes (HR = 4.269, 95% CI = 1.123–16.24) (Figure 1J). Together, high DDRGK1 protein expression levels may contribute to LUAD progression and chemotherapy resistance.

### 2.2. DDRGK1 Promotes NSCLC Cell Growth

To further investigate the functional significance of DDRGK1 in NSCLC, *DDRGK1* was knocked out in A549 and H1299 cells using the CRISPR/Cas9 system (Figure 2A). The RTCA (real-time cell analysis) migration assay showed that *DDRGK1* knockout attenuated the migratory ability of A549 and H1299 cells (Figure 2B,C). Moreover, *DDRGK1* knockout significantly decreased the anchorage-independent growth (Figure 2D,E). The effect of DDRGK1 on tumor growth was assessed using a mouse xenograft model. The average sizes and weights of tumors in the DDRGK1-knockdout group were significantly reduced compared to those in the control group (Figure 2F). To verify whether *DDRGK1* knockout was responsible for the inhibition of growth of NSCLC cells, we re-expressed DDRGK1 into *DDRGK1*-knockout cells (A549/H1299-sg*DDRGK1*-1#) (Figure S2A) and carried out a series of rescue experiments. Re-overexpression of DDRGK1 in A549/H1299-sg*DDRGK1*-1# cells enhanced cell migration (Figure S2B,C), cell anchorage-independent growth (Figure S2D,E), and xenograft tumor growth (Figure S2F) induced by *DDRGK1* knockout, suggesting that forced DDRGK1 expression could rescue the impact of sg*DDRGK1* on less tumorigenic phenotypes of A549 and H1299 cells. Together, these data demonstrate that DDRGK1 protein might play a promotional role in NSCLC progression.

### 2.3. DDRGK1 Undergoes K48-Linked Ubiquitination and Proteasome Degradation

To explore the potential mechanisms controlling DDRGK1 protein levels in NSCLC, we treated NSCLC cells with the proteasome inhibitor (MG132) and the lysosomal inhibitor (hydroxychloroquine, HCQ). Our results show that DDRGK1 protein expression was significantly upregulated after MG132 treatment, but no significant changes were observed in HCQ-treated cells (Figure 3A). Thus, we speculate that the degradation of DDRGK1 protein might be related to the ubiquitin–proteasome pathway. Then ubiquitination assay was performed to detect DDRGK1 ubiquitination under natural and denaturing conditions. The results demonstrate that DDRGK1-HA was ubiquitinated following co-expression with Flag-Ub, and this signal was further increased with MG132 treatment (Figure S3A). Subsequent analysis using linkage-specific antibodies confirmed that the polyubiquitin chains were primarily K48-linked rather than K63-linked (Figure S3B). Furthermore, stable H1299-DDRGK1-SFB cells (SFB: S-, 2×FLAG-, and Streptavidin-Binding Peptide (SBP)-tandem tags) pretreated with MG132 were used to detect DDRGK1 ubiquitination. A similar result was observed: DDRGK1-SFB underwent ubiquitination, specifically through K48-linked rather than K63-linked polyubiquitination (Figure S3C). To determine the ubiquitination of endogenous DDRGK1, H1299 cells pretreated with MG132 were lysed for IP and immunoblotting analysis, showing that endogenous DDRGK1 was modified by K48-linked ubiquitination (Figure 3B). Collectively, these results suggest that DDRGK1 could be ubiquitinated in cells, primarily through K48-linked chains.

To determine the ubiquitin E3 ligase responsible for DDRGK1 ubiquitination and degradation, we lysed 293T cells stably expressing C-terminal SFB-fused DDRGK1 protein and performed tandem affinity purification followed by mass spectrometry (TAP-MS) to screen its interacting proteins (Figure 3C). TAP-MS identified 15817 peptides for 4402 proteins, representing 2528 unique preys (Table S2). After CRAPome filtration, proteins with fold change ≥8 and P-value (Student’s *t*-test) ≤0.05 were considered to be high-confidence interacting proteins (HCIPs) (Figure S3D; Table S2). And two biological replicates showed high data repeatability (Figure S3E). Known DDRGK1-interacting proteins, such as UFL1, UFM1, UFC1, CDK5RAP3, and UFSP2, were all successfully identified (Figure 3D). To assess the reliability of our HCIPs, we compared our HCIPs with publicly available PPI records from the BioGRID database. Approximately 57% of our HCIPs were present in the BIOGRID database, indicating the specificity of our HCIPs (Figure 3E). Notably, among the 180 shared proteins, two were E3 ubiquitin ligases, namely TRIM25 and TRIM26 (Figure 3F). Then, we confirmed their interactions with DDRGK1. Co-immunoprecipitation (Co-IP) assay demonstrated that both TRIM25 and TRIM26 interacted with DDRGK1 (Figure 3G). Importantly, TRIM26, but not TRIM25, was capable of promoting K48-linked ubiquitination of DDRGK1 (Figure 3H). These results suggest that TRIM26 might be the key E3 ligase for ubiquitination and proteasomal degradation of DDRGK1.

### 2.4. TRIM26 Is a Bona Fide E3 Ligase of DDRGK1

The specific binding of an E3 ligase to its substrate constitutes the first critical event in substrate ubiquitination. To further confirm that TRIM26 functions as the E3 ubiquitin ligase of DDRGK1 and regulates its stability in NSCLC cells, we first characterized their interaction and mapped the interacting regions. Immunofluorescence staining of 293T cells overexpressing DDRGK1-HA and FLAG-TRIM26 revealed their colocalization (Figure 4A). Moreover, in NSCLC cell lines A549 and H1299, immunoprecipitation of endogenous TRIM26 co-precipitated endogenous DDRGK1 (Figure 4B), indicating an endogenous interaction between the two proteins. Further, GST-DDRGK1 and His_6_-TRIM26 proteins were purified from *E. coli* BL21 (DE3) for in vitro binding assays (Figure 4C). His_6_-TRIM26 protein could be clearly detected in GST-DDRGK1 fusion protein pulldown complex, but not in GST tag alone pulldown complex (Figure 4D), demonstrating that DDRGK1 directly interacted with TRIM26. To identify the binding regions on TRIM26 and DDRGK1, we generated a series of truncation mutants for both proteins (Figure 4E). We found that the RING and B-box domains of TRIM26 were not required for the interaction with DDRGK1-HA and that the C-terminal region (SPRY, aa 365–539) of TRIM26 was sufficient to bind DDRGK1-HA (Figure 4F). DDRGK1-HA lacking the Proteasome-CSN-eIF3 (PCI) domain (ΔPCI) fails to bind TRIM26, whereas the transmembrane (TM)-deleted mutant (ΔTM) retains binding capability (Figure 4G), indicating that the PCI domain of DDRGK1 is the critical region for binding TRIM26.

Next, we examined the effect of TRIM26 on DDRGK1 ubiquitination and degradation. Cotransfection of DDRGK1-SFB and HA-Ub with increasing amounts of Myc-TRIM26 plasmid revealed that ubiquitination of DDRGK1 was gradually enhanced by TRIM26 (Figure 5A). It has been reported that TRIM26, as an E3 ubiquitin ligase, modulates a wide range of biological processes by catalyzing K48- and K63-linked ubiquitination of its substrates, the two most common types of ubiquitin chains [26]. Thus, we examined which type of ubiquitination of DDRGK1 was triggered by TRIM26. Detection with K48- or K63-linked polyubiquitin-specific antibody revealed that TRIM26-mediated DDRGK1 ubiquitination was predominantly K48-linked ubiquitination (Figure 5A). Furthermore, more TRIM26-mediated ubiquitination of DDRGK1 was observed with a ubiquitin K48-only mutant, compared with a K63-only mutant, further supporting that TRIM26 mainly mediated K48-linked ubiquitination of DDRGK1 (Figure 5B). The co-expression of TRIM26^WT^, but not the inactive mutant (TRIM26^C16/19A^) or binding-deficient mutant (TRIM26^RBC^), promoted K48-linked ubiquitination of DDRGK-HA (Figure 5C). Further, only forced expression of TRIM26^WT^ enhanced the K48-linked ubiquitination of endogenous DDRGK1, while forced expression of TRIM26^C16/19A^ did not (Figure 5D, left panel). Correspondingly, knockdown of TRIM26 by siRNA attenuated K48-linked ubiquitination of endogenous DDRGK1 in H1299 cells (Figure 5D, right panel)). K48-linked ubiquitination primarily mediates proteasome degradation. Thus, we wonder whether TRIM26 plays a role in DDRGK1 stability. Gradient overexpression of 2Flag-TRIM26^WT^ was associated with reduced DDRGK1-HA protein abundance, yet this negative correlation was not observed in cells expressing Flag-TRIM26^C16/19A^ mutant (Figure 5E). To further determine the effect of TRIM26 on DDRGK1 degradation, cycloheximide (CHX) chase assays were conducted. The results revealed that endogenous DDRGK1 protein exhibited accelerated turnover in A549 cells stably expressing Flag-TRIM26 relative to empty vector control cells (Figure 5F,G). Taken together, TRIM26 is a bona fide E3 ubiquitin ligase for DDRGK1, which mediates its K48-linked ubiquitination and degradation.

### 2.5. TRIM26 Competes with UFL1 for Binding to DDRGK1, Facilitating Ubiquitination at K267 and Consequent Degradation

To identify TRIM26-mediated ubiquitination sites on DDRGK1, we performed pull-down assays using S-tag beads on lysates from 293T cells transiently co-expressing DDRGK1-SFB and Flag-TRIM26, followed by liquid chromatography–tandem mass spectrometry (LC-MS/MS) analysis. Six lysine residues, including K107, K116, K170, K191, K227, and K267, were identified as ubiquitinated upon co-expression of Flag-TRIM26 (Figure 6A and Figure S4A, Table S3). We then individually mutated these residues to arginines to generate DDRGK1-KR mutants and assessed their ubiquitination by TRIM26. Mutation of K267 to arginine, but not of the other residues, diminished TRIM26-mediated ubiquitination of DDRGK1 (Figure 6B). Similarly, the cancer-associated mutant K267M (according to COSMIC v102) also reduced ubiquitination of DDRGK1 by TRIM26 (Figure 6C). Functionally, the K267R mutation markedly prolonged DDRGK1 protein turnover (Figure 6D,E). Collectively, these results indicate that K267 is the primary site for TRIM26-mediated ubiquitination of DDRGK1.

Previous studies have reported K267 as a UFMylation site on DDRGK1. Our LC-MS/MS analysis validated this modification at K267 and uncovered six additional UFMylated lysine residues on DDRGK1, namely K107, K116, K157, K170, K191, and K227 (Figure 6A and Figure S4B, Table S3). However, the in vivo UFMylation assays revealed that only the K267R mutation markedly impaired the UFMylation of DDRGK1 in HEK293T-sg*UFSP2* cells, whereas mutations at other sites had no appreciable effect (Figure 6F). UFL1 is the only known E3 ligase that mediates UFMylation to date. Previous reports have reported that UFL1 regulates DDRGK1 expression in various cell lines, including HCT116 colon cancer cells, U2OS osteosarcoma cells, and HeLa cervical cancer cells [20,22]. In our study, using *UFL1*-knockout NSCLC cell lines A549 and H1299, we observed that *UFL1* depletion markedly reduced DDRGK1 protein abundance (Figure S5A). Conversely, re-expression of UFL1 significantly restored DDRGK1 protein levels (Figure S5B). Furthermore, treatment with the proteasome inhibitor MG132, but not the lysosomal inhibitor HCQ, rescued DDRGK1 expression in both *UFL1*-knockout A549 and H1299 cells (Figure S5C,D). Consistently, loss of UFL1 led to a substantial increase in K48-linked ubiquitination of endogenous DDRGK1 in H1299 cells (Figure S5E). Collectively, these findings indicate that UFL1 safeguards DDRGK1 from degradation through the ubiquitin–proteasome pathway. Accordingly, we propose that UFL1 antagonizes TRIM26-mediated ubiquitination to maintain DDRGK1 stability. As expected, the addition of UFL1-SFB and His_6_-UFM1 attenuated TRIM26-mediated ubiquitination in HEK293T-sg*UFSP2* cells, while concomitantly enhancing UFMylation of DDRGK1 (Figure 6G). Correspondingly, the addition of Flag-TRIM26 and Myc-UB increased DDRGK1 ubiquitination, whereas UFL1-mediated UFMylation was markedly reduced (Figure 6G). Given that the PCI domain mediates the binding of DDRGK1 to both UFL1 [20] and TRIM26 (Figure 4G), we propose that TRIM26 may compete for the same binding interface on DDRGK1, thereby inhibiting UFL1 interaction and consequently regulating DDRGK1 stability. To test this possibility, we performed competitive binding assays. Our results show that overexpression of Flag-UFL1 attenuated the association between DDRGK1-HA and Myc-TRIM26 (Figure 6H), whereas overexpression of Myc-TRIM26 similarly inhibited the binding of Flag-UFL1 to DDRGK1-HA (Figure 6H). Collectively, these findings are consistent with a competitive model in which TRIM26 may interfere with UFL1–DDRGK1 binding, potentially promoting ubiquitination and subsequent degradation of DDRGK1.

### 2.6. DDRGK1 Knockout Suppresses ATR Signaling and Promotes Cisplatin-Induced Apoptosis in NSCLC Cells

To elucidate the mechanism underlying the suppression of NSCLC progression by DDRGK1 downregulation, we performed GO and KEGG enrichment analyses of DDRGK1 HCIPs. The results reveal that DNA-repair-related pathways, including DNA replication, mismatch repair, and base excision repair, were mainly enriched pathways (Figure 7A; Table S4). Platinum-based drugs such as cisplatin, as the first-line chemotherapy for NSCLC, exert cytotoxicity by inducing DNA interstrand and intrastrand cross-links that block replication fork progression, and consequently give rise to DNA double-strand breaks (DSBs). DNA damage can be sensed by ATM and ATR kinases downstream, which in turn activate the damage response signaling cascade. In contrast to ATM, which responds primarily to DSBs, ATR is activated by a much wider range of genotoxic stresses. Of note, excessive ATR activation upon cisplatin has been implicated in cisplatin resistance, and its inhibition has been shown to restore cisplatin sensitivity in tumor cells [27,28]. Our mass spectrometry data revealed an interaction between DDRGK1 and ATR (Figure 3D), and the co-IP assay further confirmed that endogenous DDRGK1 interacts with ATR (Figure S6A), implying that DDRGK1 might influence DNA damage repair by modulating ATR function. To examine whether DDRGK1 was involved in the ATR pathway, we treated *DDRGK1*-knockout A549 and H1299 cells with cisplatin and examined ATR activation. We found ATR-mediated DNA damage repair response was compromised in *DDRGK1*-knockout cells, as indicated by reduced phosphorylation of ATR, as well as reduced phosphorylation of CHK1 and H2AX (Figure 7B). A decrease in γH2AX could reflect either reduced DNA damage signaling or accelerated repair, and that these two possibilities cannot be distinguished from a single time point. Thus, we performed time-course experiments following cisplatin treatment (0 h, 2 h, 4 h, and 8 h). We consistently observed that *DDRGK1* knockout suppressed γH2AX and p-ATR(T1989) accumulation at early time points (2 h) after cisplatin exposure, before substantial γH2AX clearance would occur during DNA repair (Figure S6B). This result may indicate that reduced γH2AX levels result from impaired DNA damage signaling. These findings demonstrate that DDRGK1 deficiency blunts the activation of the DNA-damage-response-associated ATR pathways.

A reduced DNA damage response is a known trigger of apoptosis. To determine whether DDRGK1 contributes to cisplatin-induced apoptosis in NSCLC, data-independent acquisition (DIA)-based quantitative proteomics was performed using *DDRGK1*-knockout A549 cells, followed by cisplatin treatment. In total, 206 differentially expressed proteins were identified relative to the control group, with 96 downregulated and 110 upregulated proteins (Figure S6C, Table S5). GO enrichment analysis of the differentially expressed proteins showed significant enrichment in the intrinsic apoptotic signaling pathway in response to DNA damage (Figure S6D); concurrently, the expression levels of pro-apoptotic proteins (HIPK1, SOD2, P53, PYCARD, etc.) were elevated (Figure S6E). In line with these findings, the protein levels of cleaved caspase-3, a marker of cell apoptosis, were significantly increased in *DDRGK1*-knockout cells upon cisplatin treatment compared to normal cells (Figure 7C). Moreover, flow cytometry with annexin V-PI staining revealed that the apoptotic rate was also higher in *DDRGK1*-knockout cells (Figure 7D and Figure S6F,G). Together, the results show that *DDRGK1* knockout augmented cisplatin-induced apoptosis in NSCLC cells.

Next, we examined the effect of *DDRGK1* knockout on cisplatin responses in NSCLC cells by assessing cell viability and clonogenic survival. *DDRGK1* knockout resulted in a significant reduction in cell viability relative to the controls (Figure 7E). Consistently, clonogenic survival assays revealed that *DDRGK1* knockout augmented the suppressive effect of cisplatin on long-term colony formation (Figure 7F and Figure S6H,I). To assess the therapeutic potential of *DDRGK1* knockout in vivo, we conducted xenograft experiments by subcutaneously injecting the A549 stable cells into nude mice. Consistent with the in vitro data, cisplatin treatment inhibited tumor growth, and *DDRGK1* knockout enhanced the inhibitory effect of cisplatin, with reduced tumor weights and volumes (Figure 7G). These results suggest that DDRGK1 modulates NSCLC response, supporting DDRGK1 as a potential therapeutic target in NSCLC.

Lastly, we determined the biological significance of DDRGK1 ubiquitination at K267 site. Compared with *DDRGK1*-knockout cells, re-expression of DDRGK1^WT^ significantly restored cisplatin-induced ATR phosphorylation, whereas the K267R mutant failed to do so (Figure 8A). Re-expression of either DDRGK1^WT^ or DDRGK1 ^K267R^ markedly reduced the levels of cleaved caspase-3 (Figure 8B) and the apoptosis rates determined via flow cytometry (Figure 8C–F), relative to the knockout controls. However, the DDRGK1^K267R^-rescued cells consistently exhibited slightly higher cleaved caspase-3 levels and apoptotic rates than the DDRGK1^WT^-rescued cells, indicating that the K267R mutant only partially recovers the anti-apoptotic function of DDRGK1. Because the K267R mutation abrogates the ubiquitin-mediated degradation and increases its protein stability, the functional discrepancy between the wild type and mutant is unlikely to result from differential protein stability. K267 is also a site for UFMylation. A more plausible interpretation is that UFMylated DDRGK1 is required for its full activity in supporting ATR signaling.

## 3. Discussion

DDRGK1 is a core protein in the UFM1 system and serves as regulator of substrate recognition and recruitment, but its regulatory mechanisms and function in NSCLC remain unclear. Here, we found that DDRGK1 was upregulated in NSCLC specimens and closely associated with malignant progression. We further identified TRIM26 and UFL1 as key factors that antagonistically govern DDRGK1 stability. UFL1 interacted with DDRGK1 to enhance its protein stability, thereby sustaining cell survival by potentiating the ATR signaling pathway. TRIM26-mediated K48-linked ubiquitination at K267 residue, which competed with UFMylation to fine-tune DDRGK1 turnover. Reduced DDRGK1 blunted cisplatin-induced ATR phosphorylation, leading to impaired DNA repair, ultimately triggering cellular apoptosis.

Several lines of evidence suggest that DDRGK1 is primarily regulated at the protein level in NSCLC. Neither the TCGA nor the CPTAC databases showed a significant difference in DDRGK1 transcript levels between normal lung and NSCLC samples (Figure 1A and Figure S1A), whereas CPTAC proteomics and our tissue microarray staining revealed higher DDRGK1 protein abundance in NSCLC samples than in normal samples (Figure 1B,D,E). This discordance may indicate that DDRGK1 protein levels are tightly regulated. We subsequently confirmed that DDRGK1 protein level is primarily controlled by the ubiquitin–proteasome pathway in cells (Figure 3A,B). We further constructed the DDRGK1 interactome via TAP-MS and identified TRIM26 as the E3 ligase that directly mediated K48-linked ubiquitination and degradation of DDRGK1 (Figure 4 and Figure 5). The K267 residue of DDRGK1 was found to subject to TRIM26-dependent ubiquitination (Figure 6A–C) and UFL1-dependent UFMylation (Figure 6A,F), suggesting a competitive interplay between these two modifications at the same residue. Competitive modification of one residue by ubiquitin and a ubiquitin-like protein is not unprecedented. For example, UFM1 conjugation stabilizes p53 by shielding it from ubiquitin-dependent degradation [22]. Notably, we also observed that UFL1 and TRIM26 compete in binding to DDRGK1 at its PCI domain, raising the possibility that the modification crosstalk at K267 may arise indirectly from this binding competition. To our knowledge, K267 of DDRGK1 is one of the first clearly documented examples where ubiquitination and UFMylation are mapped to the same lysine and shown to compete. However, the upstream signals governing the switch between these two modifications remain unclear.

Given that TRIM26 and UFL1 regulate DDRGK1 protein stability, we analyzed TRIM26 and UFL1 expression together with their correlation to DDRGK1 in LUAD and LUSC using TCGA and CPTAC datasets. The results show that *UFL1* and *TRIM26* exhibited no consistent mRNA upregulation in LUAD and LUSC (Figure S7A–C), whereas their protein abundances were significantly elevated in both subtypes (Figure S7B,C). At the mRNA level, the analysis of TCGA datasets showed a negative correlation between *UFL1* and *DDRGK1* and a positive correlation between *TRIM26* and *DDRGK1* (Figure S7D,E). The analysis of the CPTAC dataset showed no significant mRNA correlations between *DDRGK1* and either *UFL1* or *TRIM26* in LUAD or LUSC (Figure S7F,G). CPTAC proteomics showed strong positive correlation (R = 0.5001, *p* < 0.0001 in LUAD; R = 0.7644, *p* < 0.0001 in LUSC) between UFL1 and DDRGK1 protein abundance (Figure S7H), supporting that UFL1 can regulate DDRGK1 protein stability. TRIM26 protein correlated positively with DDRGK1 in CPTAC-LUSC (R = 0.2437, *p* = 0.011), but not significantly in CPTAC-LUAD (R = 0.1462, *p* =0.1275) (Figure S7I). Although TRIM26 and DDRGK1 show a positive correlation in the CPTAC-LUSC dataset, this correlation is weaker than the positive correlation observed between UFL1 and DDRGK1 (Figure S7H,I). TRIM26 itself is a regulated variable. The lack of a negative correlation between TRIM26 and DDRGK1 suggests that TRIM26-mediated degradation of DDRGK1 may depend on TRIM26 activity rather than its protein abundance.

To investigate how *DDRGK1* knockout suppresses NSCLC cell growth, the DDRGK1-interacting proteins and differentially expressed proteins from quantitative proteomic analysis of *DDRGK1*-knockout cells were subjected to enrichment analysis (Figure 7A and Figure S6D). The results show that these proteins were mainly enriched in DNA repair pathways and tightly linked to apoptosis in response to DNA damage. The interaction between DDRGK1 and ATR supported a model in which DDRGK1 is involved in regulating ATR activation. DDRGK1 knockout attenuated early γH2AX and p-ATR/p-CHK1 induction, indicating blunted DNA damage signaling. Functionally, DDRGK1 loss blunted ATR activation after cisplatin exposure and pushed cells toward apoptosis, resulting in a stronger apoptotic response to cisplatin. However, further experiments are required to clarify how DDRGK1 modulates ATR activity and to quantitatively assess DNA lesion abundance. The K267R mutation stabilizes DDRGK1, yet it activates ATR signaling less efficiently than DDRGK1^WT^ (Figure 8A). Given that K267 is also a UFMylation site, these findings indicate that UFMylation at K267 is also critical for ATR activation. UFMylation is involved in the ribosomal DNA (rDNA) damage response, where UFL1 and DDRGK1 are essential for ATM activation at nucleolar caps [23]. This raises the possibility that a related scaffolding function may operate on chromatin following replicative stress. Although our results link DDRGK1 to ATR signaling, the mechanism by which DDRGK1 supports ATR activation remains unclear. As DDRGK1 is an ER-resident, membrane-anchored protein, how it acts on nuclear ATR is unknown, pointing to a likely indirect mode of ATR regulation.

K267 lies in the C-terminal PCI domain of DDRGK1. Because PCI domains regulate protein–protein interactions, UFMylation at K267 likely serves as a conformational and interaction switch. The K267R mutation disrupts UFMylation, leading to weakened UFL1-DDRGK1 interaction [29]. K267R mutant fails to bind IRE1α, leading to IRE1α ubiquitination and degradation, and subsequent ER dysfunction and apoptosis [21,30]. In metabolic diseases, K267 modification is essential for UFBP1 to exert its protective effects against steatosis and insulin resistance [31]. Moreover, DDRGK1 UFMylation at the K267 site controls the GPCR recruitment to COPII vesicles, biosynthetic transport, and sorting at ER [32]. However, this modification is not absolutely indispensable. Ufbp1-K268R (mouse Ufbp1 K268 is the orthologous residue of human DDRGK1 K267) knockin mice are viable, fertile, and grossly normal for up to one year, exhibiting unaltered acute ER stress responses and no cardiac or intestinal phenotypes under basal or dextran sulfate sodium (DSS)-induced conditions, despite reduced total cellular UFMylation [33]. Thus, K267 UFMylation acts more as a stress- and tissue-specific amplifier that fine-tunes ligase activation and protein stability rather than as a simple on/off switch.

## 4. Materials and Methods

### 4.1. Antibodies, Reagents, Plasmids, siRNAs, and sgRNAs

Antibodies against K48-linked-specific polyubiquitin (1:1000, #8081), K63-linked-specific polyubiquitin (1:1000, #5621), p-ATR(T1989) (1:1000, #58014), ATR (1:1000, #13934), Caspase3 (1:1000, #14220S), cleaved caspase3 (1:1000, #9661S), HA-tag (1:1000, #3724), and p-CHK1(S345) (1:1000, #2348) were obtained from Cell Signaling Technology (Danvers, MA, USA). Antibodies against DDRGK1 (1:1000, 21445-1-AP), TRIM26 (1:1000, 27013-1-AP), Myc-tag (1:1000, 60003-2-lg), GST (1:1000, 66001-2-lg), β-Actin (1:1000, 60008-1-lg), and Vinculin (1:1000, 66305-2-lg) were from ProteinTech Group (Rosemont, IL, USA). UFM1 (1:1000, AB109305) antibody was from Abcam (Cambridge, UK). Ubiquitin antibody (1:1000, sc-8017) and anti-CHK1 antibody (1:1000, sc-8408) were from Santa Cruz Biotechnology (Dallas, TX, USA). Normal rabbit IgG (#A01008) was from GenScript (Nanjing, Jiangsu, China). Antibody against LC3B (1:1000, #R013940) was from Epizyme (Shanghai, China). Monoclonal anti-HA antibody (1:1000, MMS-101R) was from BioLegend (San Diego, CA, USA). Monoclonal anti-Flag M2 antibody (1:1000, F7425) and UFL1 antibody (1:1000, HPA030559) were from Sigma-Aldrich. Antibody against γH2AX (1:1000, #71197) was from GenuIN BIOTECH (Hefei, Anhui, China). Anti-HA affinity Gel (#AE059) was from Abclonal (Wuhan, China). Matrigel (#356237) was purchased from Corning (Corning, NY, USA). ECL chemiluminescence reagent (#WBKLS0500) and S-protein resin (#69704) were from Millipore (Billerica, MA, USA). Lipofectamine 2000 Transfection Reagent (#11668019) was from ThermoFisher Scientific (Waltham, MA, USA). The cell-counting kit (CCK8) (A311-02-AA) was from Vazyme (Nanjing, China). The annexin V-FITC/PI apoptosis kit (CX006L) was from Epizyme. The Anti-Flag M2 agarose affinity gel (#A2220) and Biotin (B4501) were purchased from Sigma-Aldrich (St. Louis, MO, USA). Streptavidin-conjugated beads (#17511301) were purchased from Cytiva (Marlborough, MA, USA). Protein G Plus/Protein A agarose suspension (#sc2003) was from Santa Cruz. Cisplatin (S1166) was purchased from Selleck (Houston, TX, USA).

pSG5-DDRGK1-HA was stored in our lab. DDRGK1 mutants and truncations were generated using PCR-directed mutagenesis. The cDNAs of DDRGK1, UFL1, and TRIM26 were inserted into pCDH-Flag, pCDH-Myc, or pCDH-SFB vectors for the expression of the corresponding fusion proteins. Point mutations for TRIM26 were generated using PCR-directed mutagenesis. The fragments containing the TRIM26 truncations were generated via PCR and then subcloned into pCDH-Flag vector. sgRNA against DDRGK1 or UFL1 was synthesized and cloned into the lentiCRISPR v2 vector. To create bacterial expression constructs, the fragments containing DDRGK1 and TRIM26 were subcloned into a pGEX-6p-1 and pET-28a vector, respectively. All constructs were confirmed by sequencing. The primer sequences, siRNAs, and sgRNAs used are listed in Table S6.

### 4.2. Cell Culture, Transfection, Lentivirus and Lentivirus Infection

The HEK293T cell line was purchased from ATCC (Manassas, VA, USA). The HEK293FT, A549, H1299, H23, H1975, H1944, H2126, and H838 cell lines were purchased from Wuhan Procell Biotechnology Co., Ltd. (Wuhan, China). HEK293T, HEK293FT, and A549 cells were cultured in DMEM supplemented with 10% FBS and 1% penicillin–streptomycin. H1299, H23, H1975, H1944, H2126, and H838 were cultured in RPMI-1640 containing 10% FBS and 1% penicillin–streptomycin. All cells were cultured at 37 °C in a 5% CO_2_-humidified incubator. Plasmids and siRNA transfection were carried out with PEI for HEK293T/FT, and Lipofectamine 2000 for other cells following the manufacturer’s protocol. Packaging lentiviral and subsequent infection were performed as reported previously [34]. For *DDRGK1*, *UFL1* and *UFSP2* knockout cells, single clones were isolated, cultured, and tested by immunoblotting to generate monoclonal cell lines after puromycin selection.

### 4.3. GST Pull-Down Assay

Recombinant protein expression in *E. coli* BL21(DE3) was induced through the addition of 0.2 mM isopropyl β-d-1- thiogalactopyranoside (IPTG) at 16 °C for 16 h. GST and GST-DDRGK1 proteins were purified using glutathione resins according to the manufacturer’s instructions, respectively. The recombinant His_6_-TRIM26 protein was expressed and purified from inclusion bodies using previously reported methods [34]. Purified GST-DDRGK1 was immobilized on GST beads and then incubated with or without His_6_-TRIM26 in lysis buffer (50 mM Tris-HCl pH 7.4, 300 mM NaCl, 0.5% NP-40) at 4 °C for 2 h, and the GST alone was used as the control. After washing, the bound proteins were analyzed via SDS-PAGE, followed by immunoblotting.

### 4.4. Immunoprecipitation (IP)

Cells were collected and lysed in ice-cold RIPA buffer (50 mM Tris-HCl pH 7.4, 300 mM NaCl, 1% TrionX-100, 1 mM EDTA, and 0.05% SDS) with a protease inhibitor cocktail, sonicated, and cleared by centrifugation. Lysates were incubated with Protein A/G agarose beads, and antibodies were indicated at 4 °C for 2 h. Beads were washed at least three times with RIPA buffer, and the samples were then subjected to SDS-PAGE and analyzed by immunoblotting.

For IP under denaturing conditions, the cells were lysed in SDS-lysis buffer (50 mM Tris-HCl pH 7.4, 300 mM NaCl, 1% TrionX-100, and 2% SDS) and then boiled for 10 min. Samples were diluted into 0.1% SDS with dilution buffer (50 mM Tris-HCl pH 7.4, 300 mM NaCl, 1% TrionX-100, 1 mM EDTA, and a complete protease inhibitor cocktail), and then subjected to sonication and centrifugation at 13,000 *g* for 20 min at 4 °C. The soluble supernatant fractions were harvested and subjected to IP as described above.

### 4.5. Double Immunofluorescence Staining of Tissue Microarray

An LUAD tissue microarray (ZL-LUC1601) containing 80 LUAD and 80 normal lung tissues was purchased from WEIAOBIO (Shanghai, China). The tissue microarray section was deparaffinized in xylene and rehydrated with graded ethanol solutions, followed by washing in PBS. After microwave antigen retrieval, the sections were blocked with 3% BSA for 30 min at room temperature. The sections were incubated with DDRGK1 (1:200, ProteinTech) and Ki67 (1:500, HaoKebio, Hangzhou, China) overnight at 4 °C. The tissue sections were then incubated with Flare520 (green) (HKI0014, HaoKebio) and Flare690 (pink) (HKI0017, HaoKebio) secondary antibodies for 1 h at room temperature. Nuclei were stained with DAPI (1 μg/mL) for 8 min. After washing the tissue sections with PBS, they were mounted with antifade mounting medium and imaged using a Nikon inverted-fluorescence microscope.

### 4.6. Mass Spectrometry

To identify DDRGK1-interacting proteins, SFB-tag-based TAP-MS was performed following a previously reported method [35,36]. Briefly, cell lysis from HEK293T stably expressing DDRGK1-SFB or SFB-vector were incubated with streptavidin-conjugated beads for 2 h at 4 °C. The beads were washed 3 times with NETN buffer (20 mM Tris-HCl pH 8.0, 100 mM NaCl, 1 mM EDTA, and 0.5% NP-40), and bound proteins were eluted with NETN buffer containing 2 mg/mL biotin for 2 h at 4 °C. The elutes were incubated with S-protein beads for 1 h. The beads were washed 3 times with NETN buffer and subjected to SDS-PAGE. The sample was subjected to in-gel trypsin digestion and MS by the Mass Spectrometry & Metabolomics Core Facility of Westlake University. Peptide sequences and protein identities were assigned by matching MS/MS fragmentation spectra against the Swiss-Prot protein database (Homo sapiens) using the Mascot software (Matrix Science, Boston, MA, USA) in Proteome Discoverer 2.5 (Thermo Fisher Scientific, San Jose, CA, USA). The peptide-level strict FDR threshold was set to 0.01, and the protein-level FDR threshold was set to 0.05. To identify ubiquitination sites of DDRGK1, DDRGK1-SFB was isolated using S-tag beads from HEK293T cells co-transfected with Flag-TRIM26 and Myc-Ub. To identify UFMylation sites of DDRGK1, DDRGK1-SFB was isolated using S-tag beads from HEK293T-sgUFSP2 cells co-transfected with Flag-UFL1 and HA-UFM1. The bound material was separated via SDS-PAGE and stained with Coomassie brilliant blue. Specific bands were cut out from the gel and subjected to in-gel digestion with trypsin. Then, the modified peptides were subjected to MS analysis by the Micrometer Biotech Company (Hangzhou, China).

For quantitative proteomics analysis, DDRGK1 knockout A549 cells and control cells were treated with cisplatin (20 µM) for 24 h. Cells were harvested and submitted to Nanomics Biotechnology (Hangzhou, China) for quantitative mass spectrometry analysis.

### 4.7. Migration Assay by Real-Time Cell Analysis (RTCA)

Cells were serum-starved for 6 h, trypsinized, and resuspended in serum-free medium at a density of 4 × 10^5^ cells/mL. Then, 100 μL of the suspension was seeded into the upper chamber of CIM (Cell Invasion and Migration) plate 16. Meanwhile, the lower chamber received 160 μL of complete medium containing 10% serum. The Cell Index was automatically recorded. The slopes of the time-dependent curve were analyzed using the xCELLigence software v2.0.

### 4.8. Soft-Agar Colony Formation Assay

A 2 mL aliquot of 0.7% low-melting-point agarose in complete medium with 10% serum and 1% penicillin–streptomycin was placed on the bottom of the 6-well plates as the base layer. After solidification, 1000 cells were suspended in the top agar containing 0.35% low-melting-point agarose in complete medium with 10% serum and 1% penicillin–streptomycin, and layered onto the base layer. The top agarose layer was overlaid with 3 mL of medium after it cooled and solidified. The plates were incubated at 37 °C in 5% CO_2_ for about 2-3 weeks until colonies formed. Colonies were stained with 0.005% crystal violet, photographed, and quantified using ImageJ software v1.54p.

### 4.9. Cell Viability Assay

Cells were trypsinized, counted, and resuspended at a density of 5 × 10^4^ cells/mL. Then, 100 μL of the suspension was seeded in a 96-well plate in replications. After 6 h of incubation, cisplatin at the indicated concentrations was added and cultured for another 48 h. Cell viability was determined using the CCK-8 kit by measuring OD450, and the values were normalized to those of the non-treated group.

### 4.10. Plate Colony Formation Assay

Cells were seeded in 6-well plates at 2000 cells per well. After 3 days, 0.5 μM of cisplatin was added. The medium containing the indicated drugs was refreshed every 3 days until colony was visible. Colonies were washed, fixed, stained with 1% crystal violet overnight, and quantified using ImageJ.

### 4.11. Annexin V-FITC/PI Staining for Apoptosis

A549/H1299 stable cells were seeded in 6 cm dishes. After 24 h, the cells were treated with 20 μM of cisplatin for another 48 h. Subsequently, Annexin V-FITC/PI staining was performed as described in the guidelines of the assay kit. Briefly, 100 μL of cell suspension (1 × 10^5^ cells) was mixed with 5 μL of Annexin V-FITC and 10 μL of PI and incubated in the dark at room temperature for 10–15 min. After that, the cells were centrifuged and re-suspended in 100 μL of binding buffer for immediate flow cytometric analysis.

### 4.12. Xenograft Tumor Model

Six-week-old male BALB/c nude mice were randomly divided into indicated groups. A549 stable cells in 60 µL of Opti-MEM medium containing 5 × 10^6^ cells were mixed with an equal volume of Matrigel and injected subcutaneously into the flanks of the nude mice. Three independent xenograft experiments were conducted, with group sizes of *n* = 8, *n* = 5, and *n* = 6 mice per group, respectively. All mice were sacrificed at about 30 days, and tumors were photographed and weighed. To investigate the effect of DDRGK1 knockout on the therapeutic role of cisplatin, the mice were randomly divided into groups (*n* = 6 per group) at 14 days after injection. The mice were treated with or without cisplatin (5 mg/kg) by intraperitoneal injection every 3 days, with 5 injections in total. The mice were sacrificed on day 3 after the final treatment, and the tumors were photographed and weighed. All animal studies were performed in compliance with the ARRIVE 2.0 guidelines and conducted with the approval and guidance of Hangzhou Normal University Medical Animal Ethics Committees.

### 4.13. Gene and Protein Expression Database Analysis

The *DDRGK1* mRNA expression data of the TCGA-LUAD and TCGA-LUSC cohorts were obtained from UCSC Xena (GDC hub, accessed on 06 Oct 2023). The DDRGK1 mRNA expression and protein expression data of CPTAC-LUAD and CPTAC-LUSC were obtained from the Clinical Proteomic Tumor Analysis Consortium (CPTAC) (data were retrieved via a LinkedOmics portal, accessed on 10 Oct 2023). Genomic and dependency data were obtained from the DepMap portal (26Q1, accessed on 05 Jul 2026) Statistical analyses were performed with GraphPad Prism 10.

### 4.14. Quantification and Statistical Analysis

All data are presented as the mean ± standard deviation (SD) unless otherwise indicated. Statistical analyses were performed with GraphPad Prism 10. Student’s t-test, one-way ANOVA, and two-way ANOVA were used for group comparisons. For analyses with small sample sizes, these parametric tests were applied under the assumptions of normally distributed residuals and homoscedasticity. Formal normality and variance homogeneity tests were not performed because small sample sizes lack sufficient statistical power to yield reliable outcomes. *p* < 0.05 was defined as statistically significant.

## 5. Conclusions

DDRGK1 protein is upregulated in NSCLC and correlated with poor prognosis. DDRGK1 levels are regulated by TRIM26-mediated ubiquitination, whereby TRIM26 competes with UFL1 for binding to DDRGK1, facilitating ubiquitination at K267 and consequent degradation. *DDRGK1* knockout reduces ATR phosphorylation and augments cisplatin-induced apoptosis in NSCLC cells both in vitro and in vivo.

## Figures and Tables

**Figure 1:**
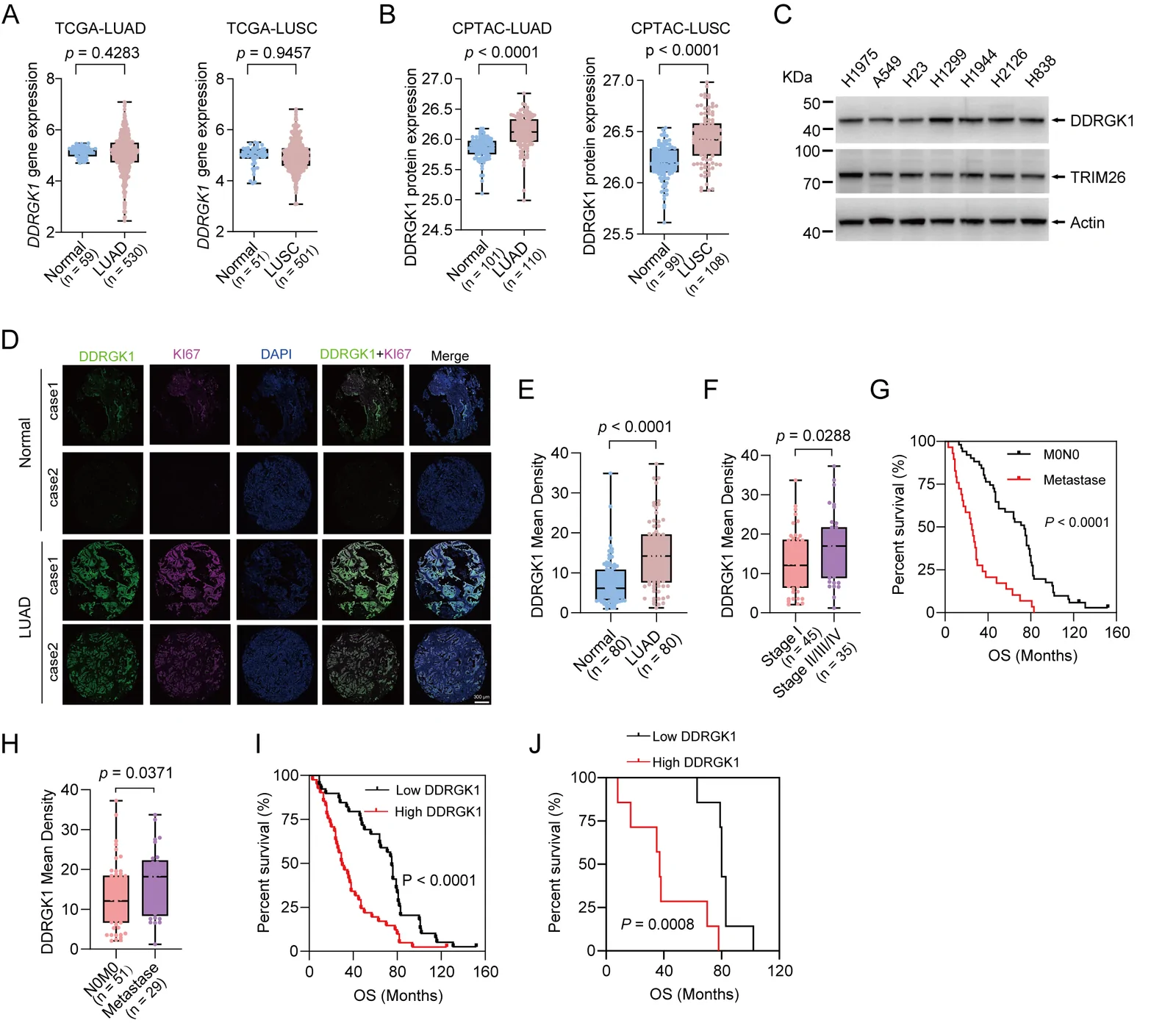
Clinical impact of DDRGK1 expression on NSCLC progression. (**A**) Analysis of *DDRGK1* mRNA expression levels in LUAD/LUSC tissues and normal lung tissues from TCGA database. (**B**) Protein levels of DDRGK1 were increased in LUAD and LUSC. Analysis of DDRGK1 protein expression levels in LUAD/LUSC tissues and normal lung tissues from CPTAC database. (**C**) The protein expression levels of DDRGK1 in non-metastatic (H1975, A549, and H23) and metastatic (H1299, H1944, H2126, and H838) NSCLC cell lines. (**D**) Double immunofluorescence staining of DDRGK1 and Ki67 in human normal lung (*n* = 80) and LUAD (*n* = 80) tissues. Representative images were shown. Scale bars: 300 μm. (**E**) Statistical analysis of DDRGK1 immunofluorescence staining intensity in human LUAD tissue microarray. (**F**) Quantification of DDRGK1 immunofluorescence staining intensity in early-stage (stage I, *n* = 45) and advanced-stage (stages II/III/IV, *n* = 35) LUAD tissues. (**G**) Kaplan–Meier curves of overall survival in LUAD patients with and without metastasis. n (M0N0) = 51, n (Metastase = 29). (**H**) Quantification of DDRGK1 immunofluorescence staining intensity in non-metastatic (*n* = 51) versus metastatic (*n* = 29) LUAD tissues. (**I**) Kaplan–Meier curves for overall survival in LUAD patients (*n* = 80) stratified by DDRGK1 expression, dichotomized at the median into high (above median, *n* = 40) and low (below median, *n* = 40) groups. (**J**) Kaplan–Meier curves for overall survival in LUAD patients who received chemotherapy (*n* = 14), stratified by DDRGK1 expression, dichotomized at the median into high (above median, *n* = 7) and low (below median, *n* = 7) groups.

**Figure 2:**
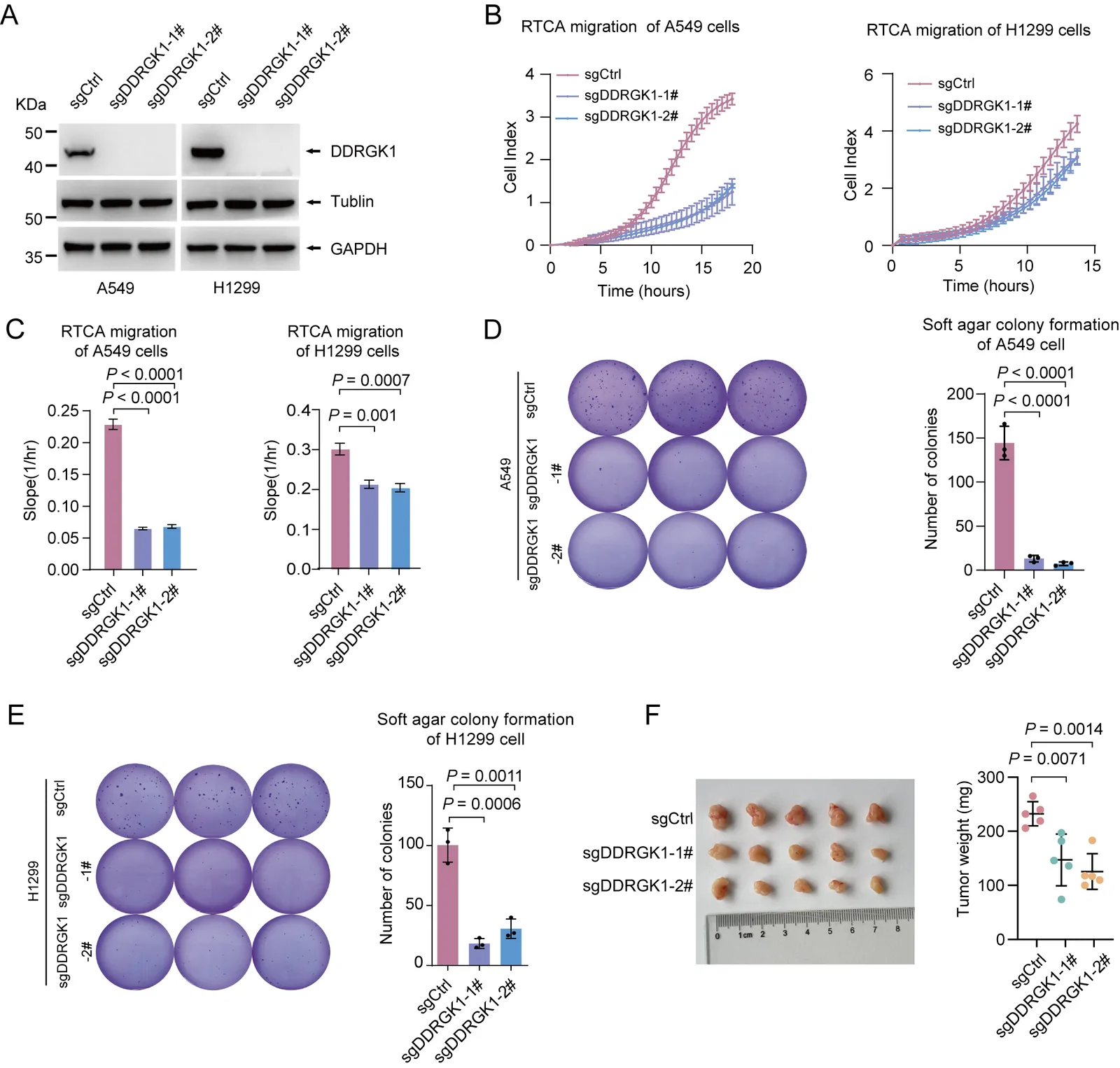
*DDRGK1* knockout suppresses NSCLC cell progression. (**A**) The efficiency of *DDRGK1* knockout in A549 and H1299 cells. The levels of DDRGK1 protein were determined by immunoblotting analysis. (**B**) Cell migration was assessed using the real-time cell analysis (RTCA) xCELLigence system. *DDRGK1* knockout A549 and H1299 stable cells were seeded into a CIM-Plate and subjected to a dynamic migration assay, respectively. Error bars indicate ± SD. (**C**) Bar chart showing the migration slopes in B (mean ± SD; *n* = 3 independent biological experiments; Student’s *t* test). (**D**,**E**) *DDRGK1* knockout A549 and H1299 stable cells were tested for anchorage-independent growth in a soft-agar colony assay. Viable colonies after about 2 weeks were counted (mean ± SD; *n* = 3 independent biological experiments; one-way ANOVA). (**F**) The indicated stable A549 cells were subcutaneously injected into 6-week-old male BALB/c nude mice. Tumors were harvested, and the tumor weight was calculated (mean ± SD; *n* = 5 independent biological experiments; one-way ANOVA).

**Figure 3:**
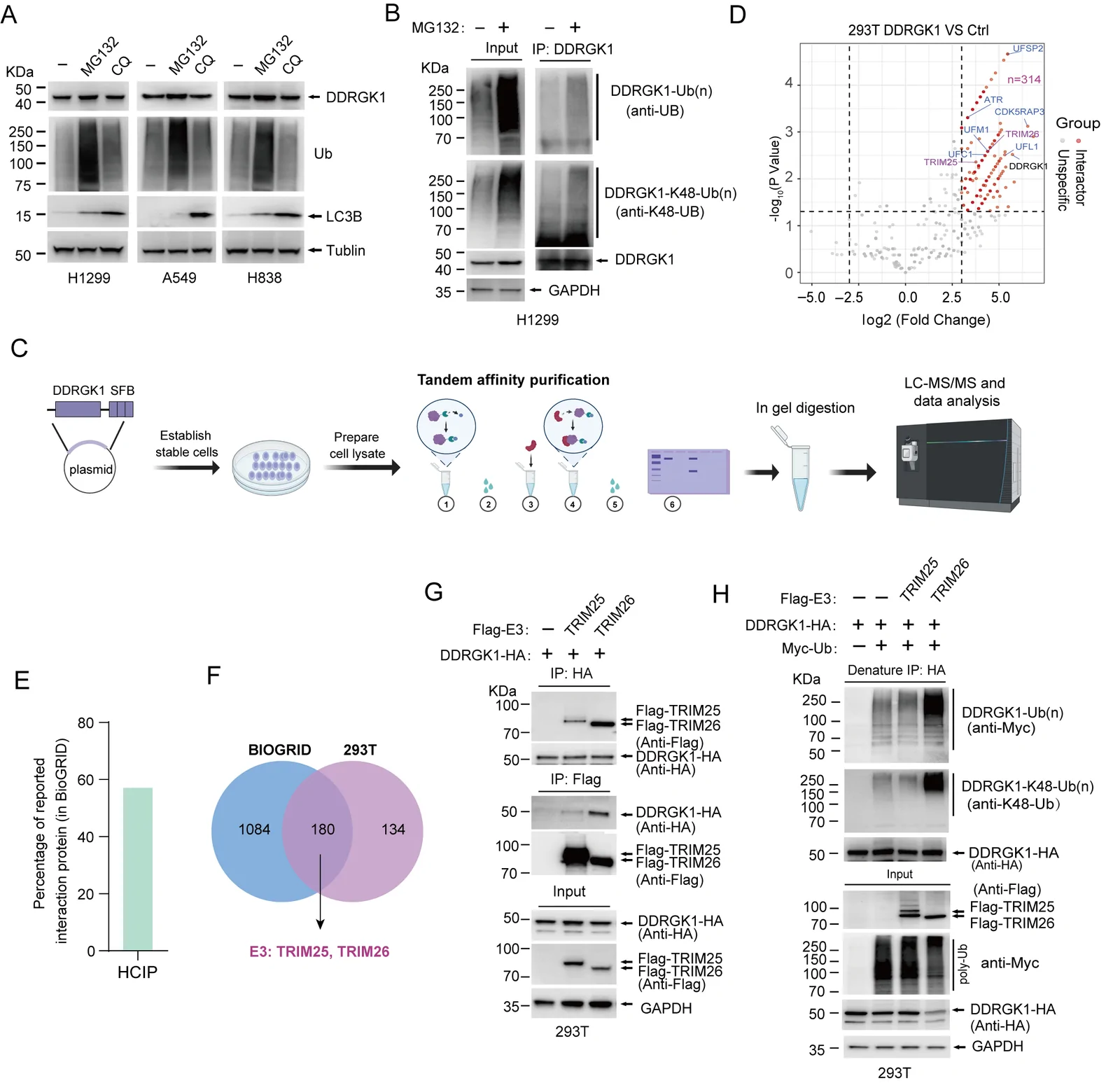
The identification of DDRGK1-interacting proteins. (**A**) Cellular DDRGK1 was predominantly degraded through the proteasome. NSCLC cell lines A549, H1299, and H838 were treated with MG132 (20 μM, 8 h) or HCQ (10 μM, 8 h) followed by immunoblotting analysis using the indicated antibodies. (**B**) DDRGK1 underwent K48-linked ubiquitination. Lysates from H1299 cells treated with or without MG132 (20 μM, 8 h) were immunoprecipitated with anti-DDRGK1 antibody, followed by immunoblotting with the indicated antibodies. (**C**) Workflow for proteomic identification of DDRGK1-binding proteins. Figures were created with BioRender Guo, G. (2026) https://BioRender.com/1ojnum9). (**D**) Volcano plot showing the HCIPs of DDRGK1. (**E**) Percentages of reported interactions in HCIPs. (**F**) Venn diagram-based analysis of DDRGK1-interacting proteins from our identified HCIPs with the existing PPI databases of BioGRID. Two ubiquitin E3 ligases, TRIM25 and TRIM26, were common to both datasets. (**G**) Lysates from HEK293T cells co-expressing DDRGK1-HA with Flag-TRIM25 or Flag-TRIM26 were subjected to Co-IP and immunoblotting analysis with indicated antibodies. (**H**) TRIM26 conjugated K48-linked polyubiquitin chains to DDRGK1. Co-transfected HEK293T cells indicated that the plasmids were lysed for IP under denaturing conditions, and ubiquitination of DDRGK1 was detected by anti-ubiquitin and K48-linked-specific polyubiquitin antibodies.

**Figure 4:**
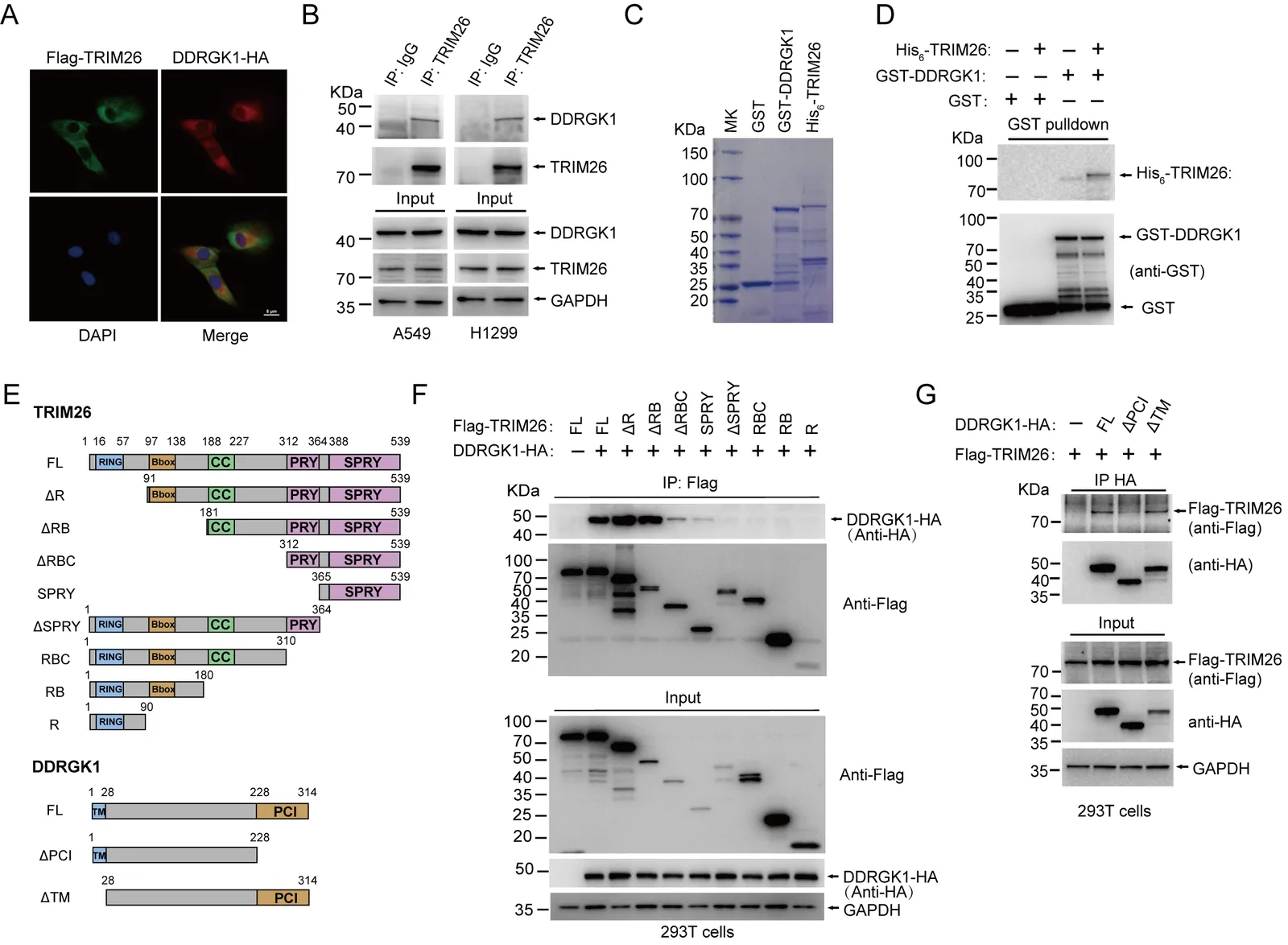
DDRGK1 directly interacted with TRIM26. (**A**) Immunofluorescence staining of 293T cells co-transfected with DDRGK1-HA and Flag-TRIM26. Scale bars: 5 μm. (**B**) Endogenous TRIM26 was immunoprecipitated from A549 and H1299 cells, followed by immunoblotting with an antibody against DDRGK1. (**C**) GST, GST-DDRGK1, and His_6_-TRIM26 proteins purified from *E. coil* BL21 (DE3) were analyzed via coomassie brilliant blue staining. (**D**) Purified GST and GST-DDRGK1 were co-incubated with or without His_6_-TRIM26 for GST pull-down assay. The precipitated material was subjected to immunoblotting analysis. (**E**) Overview of TRIM26 and DDRGK1 truncations. (**F**) Lysates from HEK293T cells transfected with DDRGK1-HA and Flag-tagged TRIM26 truncations were immunoprecipitated with anti-FLAG M2 beads, followed by immunoblotting with anti-HA antibody. (**G**) Lysates from HEK293T cells transfected with Flag-TRIM26 and HA-tagged DDRGK1 truncations were immunoprecipitated with HA beads, followed by immunoblotting with anti-Flag antibody.

**Figure 5:**
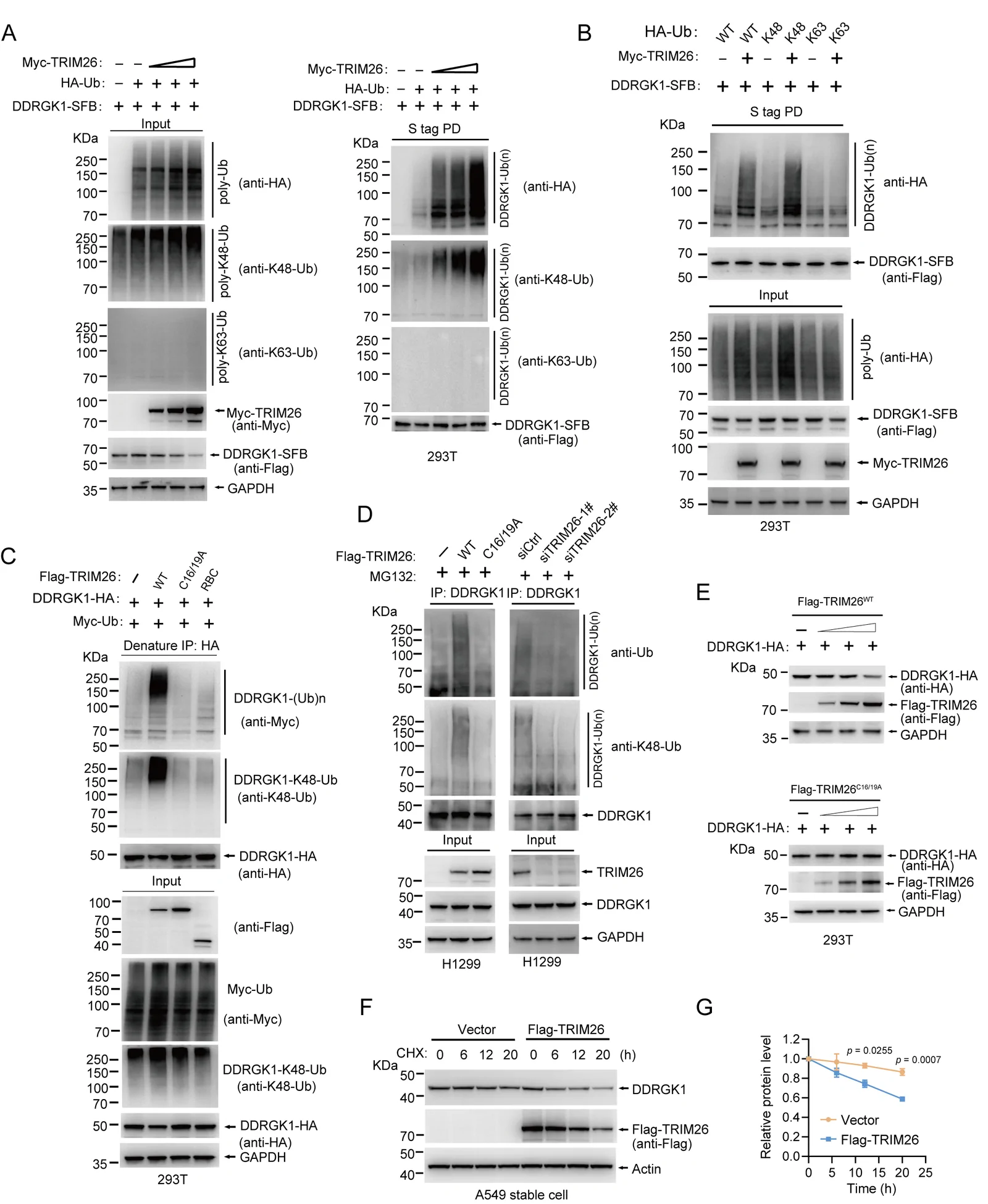
TRIM26 mediated DDRGK1 K48-linked ubiquitination and degradation. (**A**) Lysates from HEK293T cells transfected with DDRGK1-SFB and HA-Ubiquitin together with an increasing amount of Myc-TRIM26 were pulled down with S-tag beads, followed by immunoblotting analysis with the indicated antibodies. (**B**) Lysates from HEK293T cells co-expressing DDRGK1-SFB and Myc-TRIM26 with HA-Ubiquitin wild type (WT) or its K-only mutant (Ub-K48/K63) were pulled down with S-tag beads, followed by immunoblotting analysis with anti-HA antibody. (**C**) Lysates from HEK293T cells co-transfected DDRGK1-HA, Myc-Ub together with Flag-TRIM26^WT^, inactive mutant Flag-TRIM26^C16/19A^, or binding-deficient mutant Flag-TRIM26 ^RBC^ were immunoprecipitated with HA beads under denaturing condition, followed by immunoblotting analysis with the indicated antibodies. (**D**) H1299 cells were transfected with TRIM26^WT^, TRIM26^C16/19A^ or TRIM26 siRNAs and then treated with 20 μM MG132 for 8 h. Endogenous DDRGK1 was immunoprecipitated from cell lysates, followed by immunoblotting analysis with the indicated antibodies. (**E**) Lysates from HEK293T cells transfected with DDRGK1-HA and an increasing amount of Flag-TRIM26 were used for immunoblotting with the indicated antibodies. (**F**) The relative turnover of endogenous DDRGK1 protein was determined in Flag-TRIM26 overexpressing A549 cells with CHX (100 μg/mL) treatment for the indicated time points. (**G**) The DDRGK1 band intensities in F were quantitated (mean ± SD; *n* = 3 independent biological experiments; two-way ANOVA). All of the above experiments were repeated at least 3 times.

**Figure 6:**
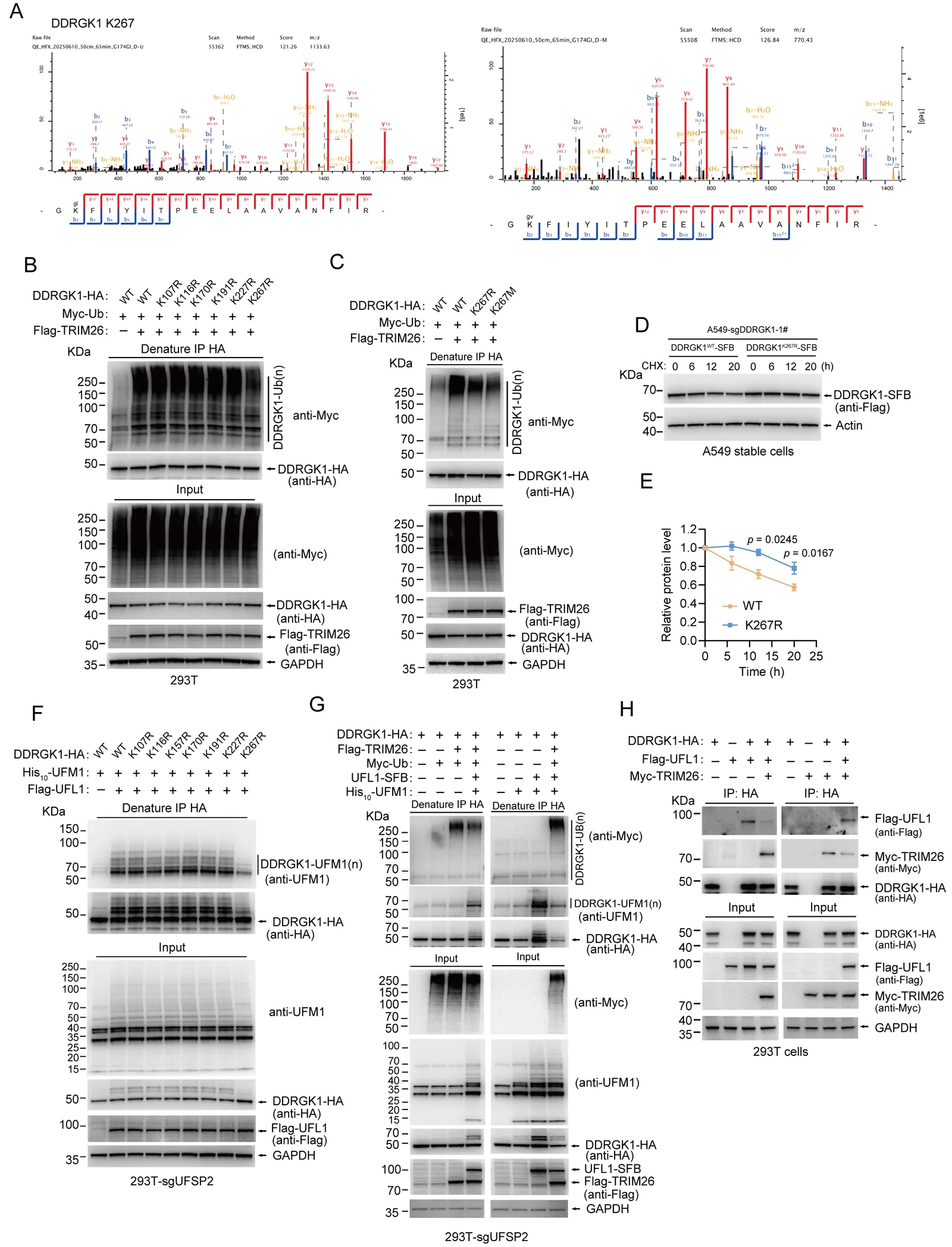
Competitive ubiquitination and UFMylation at K267 controls DDRGK1 stability. (**A**) Identification of the ubiquitination and UFMylation sites in DDRGK1 by mass spectrometry. Tandem mass spectrum of peptides showed ubiquitin (left) and UFM1 (right) conjugation at the K267 residue of DDRGK1. (**B**,**C**) Lysates from HEK293T cells co-transfected with DDRGK1-HA mutant (K107R, K116R, K170R, K191R, K227R, K267R, or K267M), and the indicated plasmids were immunoprecipitated with anti-HA antibody under denaturing condition, followed by immunoblotting analysis with anti-Myc antibody. (**D**) Determination of DDRGK1 relative turnover in *DDRGK1* knockout A549 cells stably reconstituted with DDRGK1^WT^-SFB and DDRGK1^K267R^-SFB, following CHX (100 μg/mL) treatment for the indicated time points. (**E**) The DDRGK1 band intensities in D were quantitated (mean ± SD independent biological experiments; *n* = 3; two-way ANOVA). (**F**) Lysates from *UFSP2* knockout HEK293T cells co-transfected with DDRGK1-HA mutant (K107R, K116R, K157R, K170R, K191R, K227R, or K267R), and the indicated plasmids were immunoprecipitated with anti-HA antibody under denaturing condition, followed by immunoblotting analysis with anti-UFM1 antibody. (**G**) Lysates from *UFSP2* knockout HEK293T cells co-transfected with the indicated plasmids were immunoprecipitated with anti-HA antibody under denaturing conditions, followed by immunoblotting analysis with the indicated antibodies. (**H**) Lysates from HEK293T cells co-transfected with indicated plasmids were immunoprecipitated with anti-HA antibody, followed by immunoblotting analysis with the indicated antibodies. Experiments were repeated at least 3 times.

**Figure 7:**
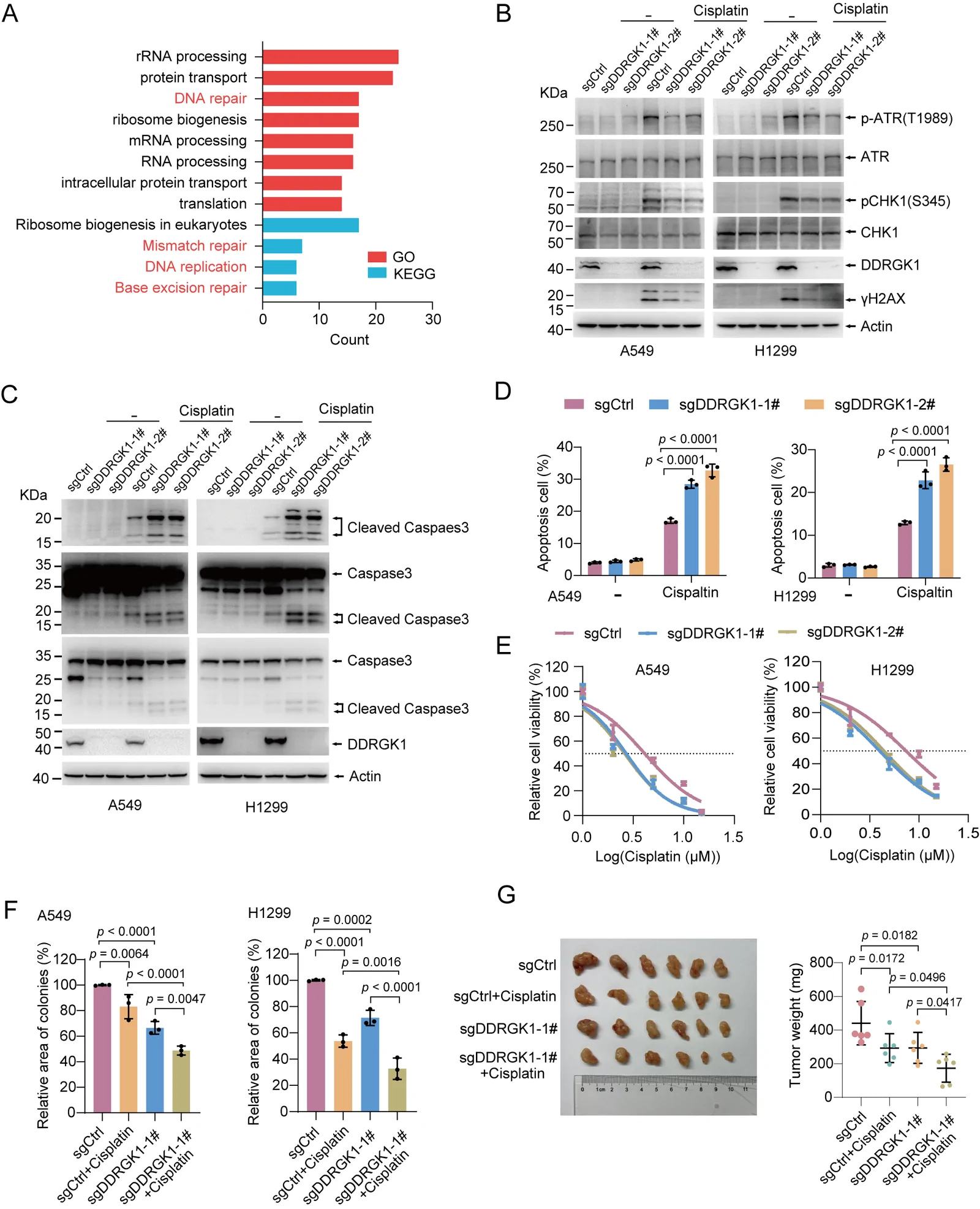
*DDRGK1* knockout increases the cytotoxic effect of cisplatin in NSCLC cells. (**A**) Gene Ontology (GO) and KEGG enrichment analysis of the 314 HCIPs of DDRGK1. DNA repair-related enriched pathways are highlighted in red. (**B**) *DDRGK1* knockout A549 and H1299 cells were treated with cisplatin (20 μM, 12 h), and cell lysates were harvested for immunoblotting analysis. (**C**) *DDRGK1* knockout A549 and H1299 cells were treated with cisplatin (20 μM, 48 h), and cell lysates were harvested for immunoblotting analysis. (**D**) *DDRGK1* knockout A549 and H1299 cells were treated with cisplatin (20 μM, 48 h), and cells were harvested for Annexin V-FITC/PI staining and flow cytometry analysis (mean ± SD independent biological experiments; *n* = 3 independent biological experiments; two-way ANOVA). (**E**) Cell viability was assessed by CCK8 assay in *DDRGK1* knockout A549 and H1299 cells at 48 h after cisplatin treatment. In A549 cells, the cisplatin IC50 was 4.10 μM (sgCtrl), 2.70 μM (sgDDRGK1-1#), and 2.60 μM (sgDDRGK1-2#) (*p* = 0.0002; F (4,54) = 6.538; extra sum-of-squares F test). In H1299 cells, the IC50 was 7.38 μM (sgCtrl), 3.98 μM (sgDDRGK1-1#), and 4.41 μM (sgDDRGK1-2#) (p < 0.0001; F (4,54) = 12.52; extra sum-of-squares F test). Data are presented as mean ± SD, *n* = 4 independent biological experiments. (**F**) The plate colony formation detection clonogenic ability of *DDRGK1* knockout A549 and H1299 cells under cisplatin treatment (mean ± SD; *n* = 3 independent biological experiments; two-way ANOVA). (**G**) The *DDRGK1* knockout A549 cells and control cells were subcutaneously injected into 6-week-old male BALB/c nude mice. At 14 days after injection, mice received intraperitoneal injections of cisplatin (5 mg/kg) or saline every 3 days, with 5 injections in total. Tumors were harvested, and the tumor weight was calculated (mean ± SD; *n* = 6 independent biological experiments; two-way ANOVA).

**Figure 8:**
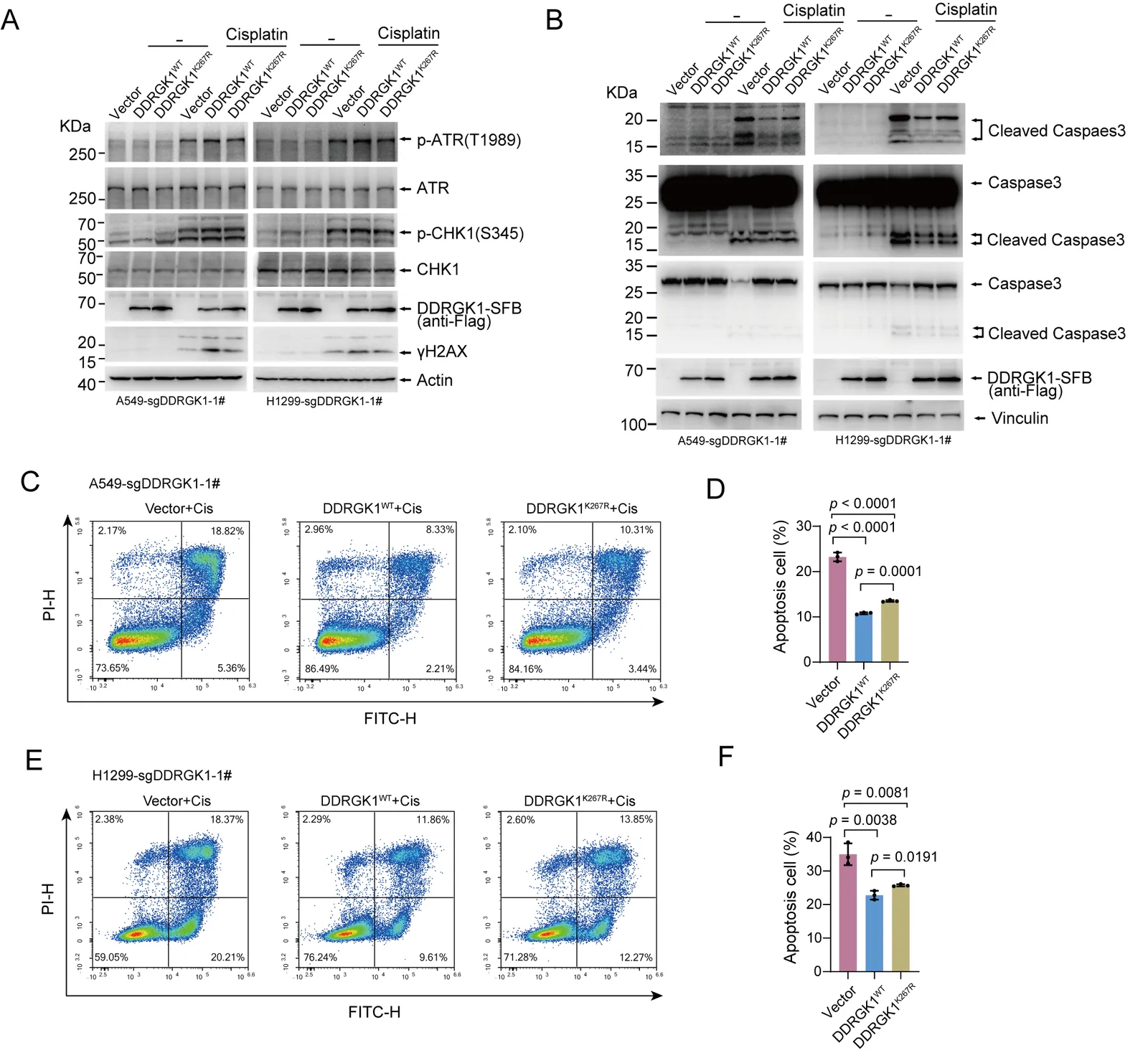
K267R mutant impaired the function of DDRGK1 in regulating ATR signaling and apoptosis. (**A**) *DDRGK1*-knockout A549 and H1299 cells were stably reconstituted with an empty vector, DDRGK1^WT^ or DDRGK1^K267R^ were treated with cisplatin (20 μM, 12 h), and cell lysates were analyzed with the indicated antibodies. (**B**) *DDRGK1*-knockout A549 and H1299 cells were stably reconstituted with an empty vector, DDRGK1^WT^ or DDRGK1^K267R^ were treated with cisplatin (20 μM, 48 h), and cell lysates were harvested for cleaved caspase3 analysis. (**C**–**F**) *DDRGK1*-knockout A549 (**C**,**D**) and H1299 (**E**,**F**) cells were stably reconstituted with an empty vector, DDRGK1^WT^ or DDRGK1^K267R^ were treated with cisplatin (20 μM, 48 h), and cells were harvested for Annexin V-FITC/PI staining and flow cytometry analysis. D and F show the quantification of C and E, respectively (mean ± SD; *n* = 3 independent biological experiments; Student’s *t* test).

## Data Availability

The original contributions presented in this study are included in the article/Supplementary Material. Further inquiries can be directed to the corresponding author.

## Supplementary Materials

The following supporting information can be downloaded at https://www.mdpi.com/article/10.3390/ijms272110014/s1.
