# Supplementary material for: TRIM26-Mediated Ubiquitination and Degradation of DDRGK1 Suppress NSCLC Progression by Inhibiting DNA Repair and Promoting Apoptosis
Source: Int J Mol Sci. 2026 Nov 8;27(21):10014. doi: 10.3390/ijms272110014 (PMC13763867; doi:10.3390/ijms272110014)
Supplement: Supplementary file 1 [file ijms-27-10014-s001.zip › ijms-4649086 Supplement clear.pdf]

TRIM26-mediated ubiquitination and degradation of DDRGK1 suppress NSCLC progression by inhibiting DNA repair and promoting apoptosis

Yanmin Guo[1], Qifan Guo[1], Jingjin Yu[1], Yu Cao[1], Dexin Rao[1], Ziruo Guo[1], Binglong Li[1], Fanjian Jia[1], Qian Liang[1], Yang Zhuang[1], Shiwen Xu[1], Yaxiang Wang[1], Miao Wang[1], Yu-Sheng Cong[1] ✉

[1]Zhejiang Key Laboratory of Medical Epigenetics, Key Laboratory of Aging and Cancer Biology of Zhejiang Province, Hangzhou Normal University School of Basic Medical Sciences, Hangzhou, 311121, China

✉ **Correspondences: Dr. Yu-Sheng Cong,**

yscong@hznu.edu.cn

Supplementary Figures

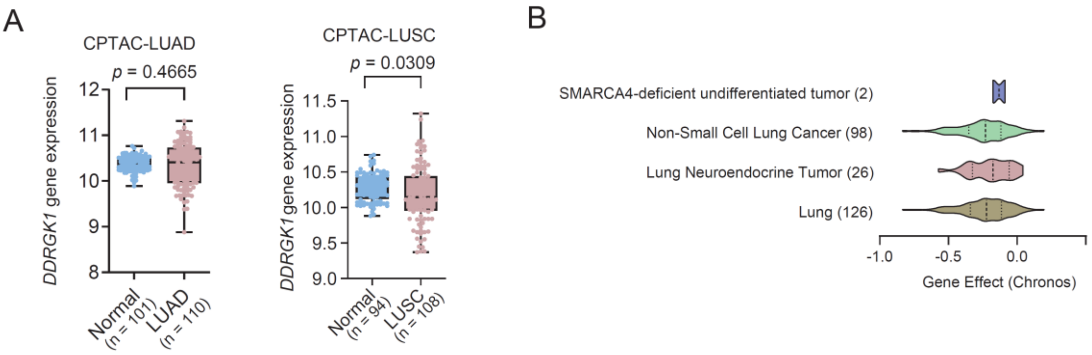

Figure S1 Clinical impact of DDRGK1 expression on NSCLC progression. (A) Analysis of *DDRGK1* mRNA expression levels in LUAD/LUSC tissues and normal lung tissues from Clinical Proteomic Tumor Analysis Consortium (CPTAC) database. (**B)** The gene dependency scores analysis of 126 lung cancer cell lines obtained from the DepMap database. Negative dependency scores for DDRGK1 reveal that *DDRGK1* knockout suppresses tumor cell growth.

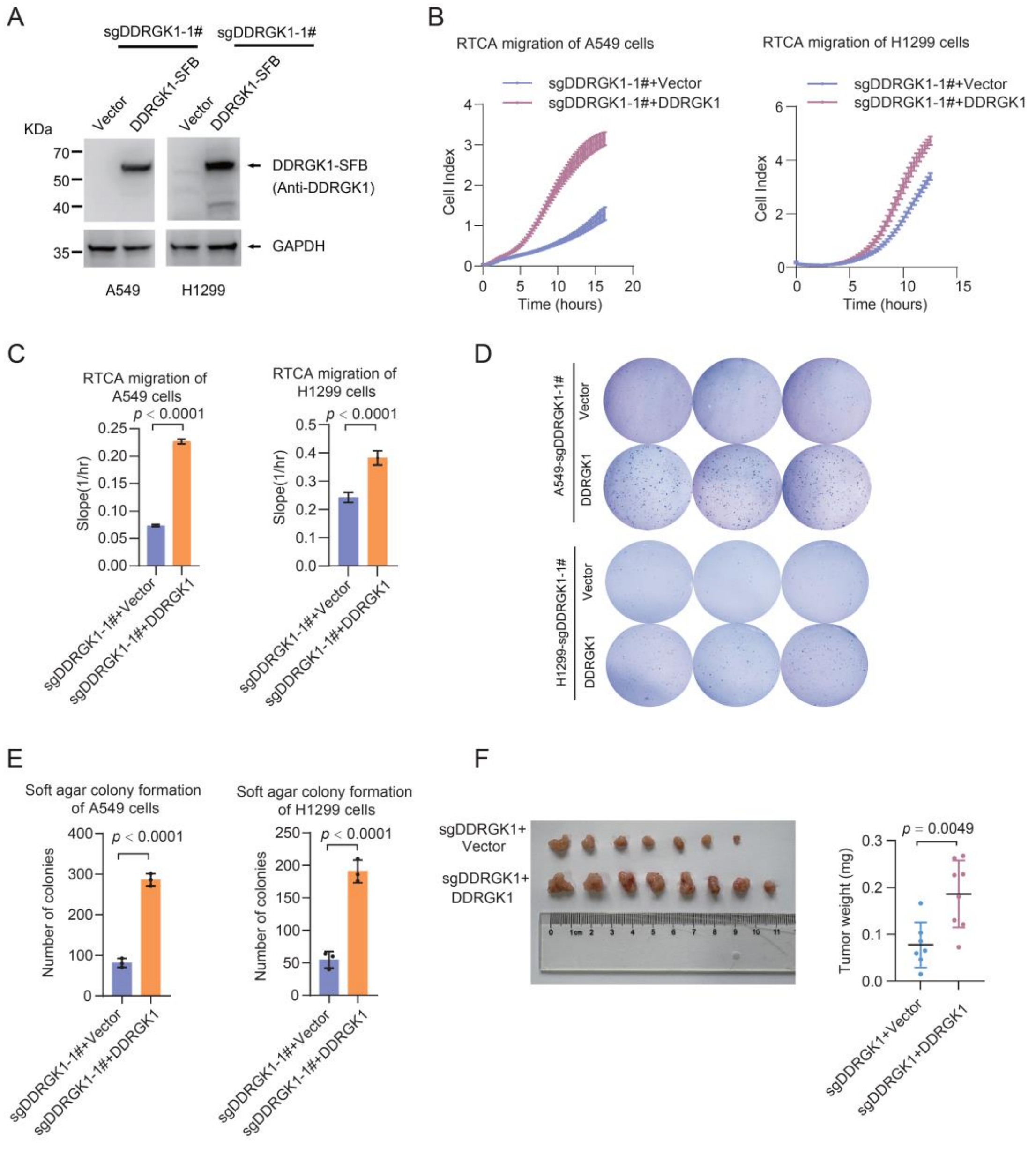

Figure S2 Reconstituted expression of DDRGK1 promotes NSCLC cell progression. (A) Immunoblotting was performed to verify the re-expression of DDRGK1 in *DDRGK1*-knockout A549 and H1299 cells. (**B)** Effect of DDRGK1 reconstitution on the migration was assessed using the real-time cell analysis (RTCA) xCELLigence system as described in method. Error bars indicate ± SD. **(C)** Bar chart showing the migration slopes in B (Mean ± SD; n = 3 independent biological experiments; Student's *t* test). **(D-E)** Soft-agar colony formation assays for stable re-expression of DDRGK1 in *DDRGK1*-knockout A549 and H1299 cells. Colonies were photographed (D) and then quantified (Mean ± SD; n = 3 independent biological

experiments; Student's *t* test) (E). (**F**) *DDRGK1*-knockout A549 cells stably reconstituted with DDRGK1 or empty vector were subcutaneously injected into BALB/c nude mice. Tumors were harvested and the tumor weight was calculated (Mean ± SD; n = 7 (upper) and n = 8 (lower) independent biological experiments; Student's *t* test).

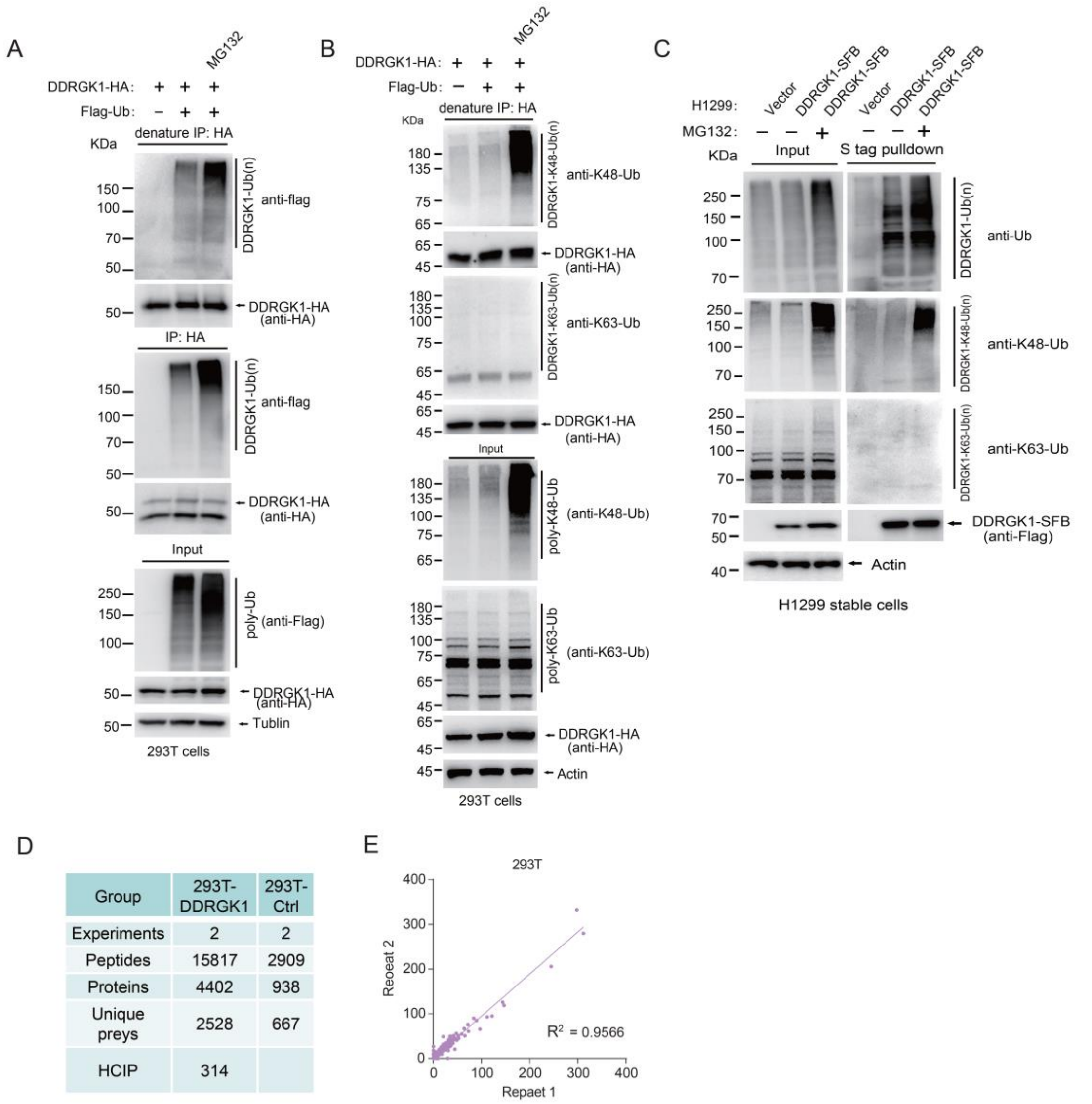

Figure S3 DDRGK1 underwent K48-linked ubiquitination. (A-B) HEK293T cells transfected with DDRGK1-HA and Flag-Ubiquitin were treated with or without MG132 (20 μM, 8 h). The lysates were harvested for IP with HA beads under naturing or denaturing condition, and followed by immunoblotting analysis with indicated antibodies to detect DDRGK1 ubiquitination and the ubiquitin chain linkage types. **(C)**

H1299 cells stably expressing DDRGK1-SFB were treated with or without MG132 (20 μM, 8 h). The lysates were harvested for pulldown with S-tag beads, and followed by immunoblotting analysis with indicated antibodies to detect DDRGK1 ubiquitination and the ubiquitin chain linkage types. **(D)** Data summary of DDRGK1-interacting proteins identified by TAP-MS. The total numbers of peptides and proteins identified in the MS analysis are shown. High-confidence interacting proteins (HCIPs) were identified using Fold change $\geqslant 8$ and P-value (Student's t-test) $\leqslant 0.05$. **(E)** Data reproducibility for the TAP-MS of DDRGK1. Two biological repeats of MS analysis were performed. Identified preys were used to estimate the data reproducibility at the protein levels.

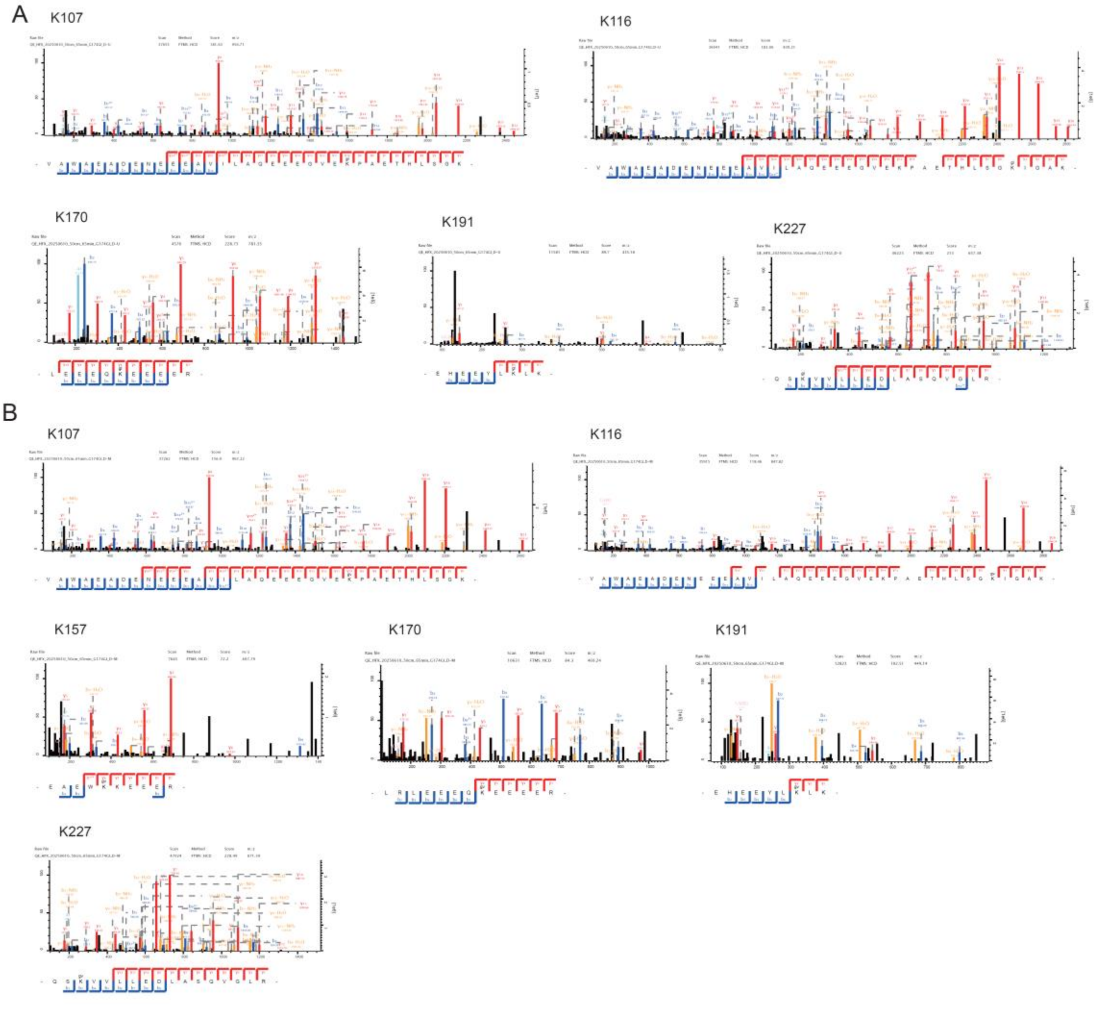

Figure S4 Mass spectrometry analysis of the potential ubiquitination and UFMylated sites in DDRGK1. (A) Tandem mass spectrum of peptides showed

ubiquitin conjugation at the K107R, K116R, K170R, K191R and K227R residues of DDRGK1. (**B)** Tandem mass spectrum of peptides showed UFM1 conjugation at the K107R, K116R, K157R, K170R, K191R and K227R residues of DDRGK1. Supplemental data for Figure 6A.

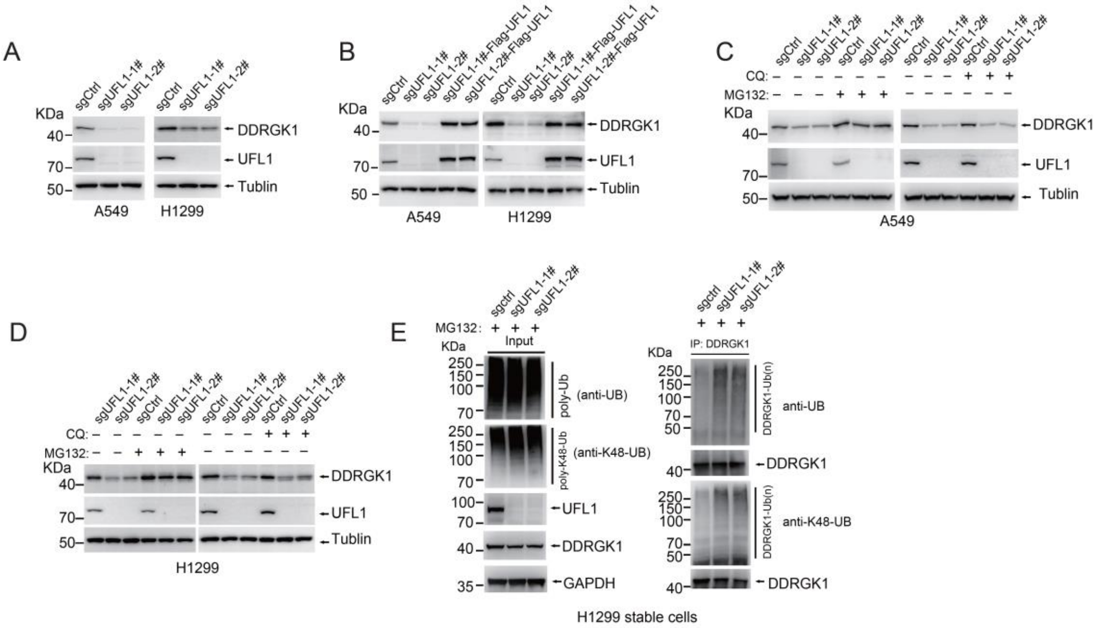

Figure S5 ***UFL1*** **knockout promoted DDRGK1 ubiquitination and degradation. (A)** The efficiency of *UFL1* knockout in A549 and H1299 cells. The levels of DDRGK1 protein were determined by immunoblotting analysis. (**B)** *UFL1*-knockout A549 and H1299 cells stably reconstituted with UFL were lysed for immunoblotting analysis with indicated antibodies. (**C-D)** *UFL1* knockout A549 (C) and H1299 (D) cells were treated with MG132 (20 μM, 8 h) or chloroquine HCQ (10 μM, 8 h) followed by immunoblotting analysis with indicated antibodies. (**E)** *UFL1* knockout H1299 cells were treated with 20 μM MG132 for 8 h. Endogenous DDRGK1 was immunoprecipitated from cell lysates and followed by immunoblotting analysis with indicated antibodies.

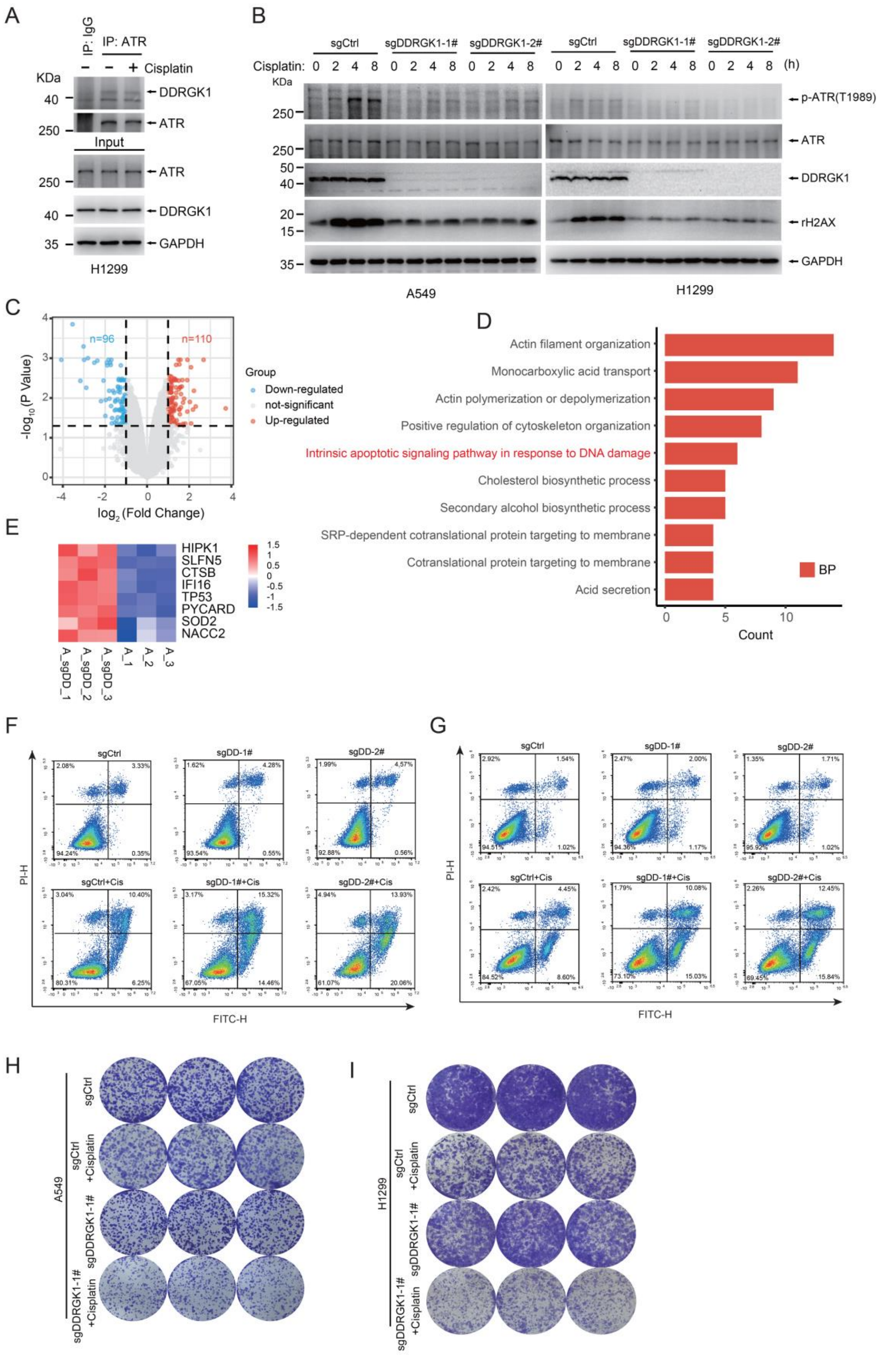

**Figure S6 *DDRGK1* knockout suppresses NSCLC cell survival by inhibiting DNA

damage repair and promoting apoptosis upon cisplatin treatment. (A) Endogenous ATR was immunoprecipitated from H1299 cells treated with or without cisplatin (20 μM, 12 h) and followed by immunoblotting with antibody against DDRGK1. **(B)** *DDRGK1* knockout A549 and H1299 cells were treated with 20 μM cisplatin for indicated time and cell lysates were harvested for immunoblotting analysis. **(C)** *DDRGK1* knockout A549 cells and control cells were treated with cisplatin (20 μM) for 24 h. Cells were harvested for quantitative proteomics analysis. Volcano plot for differentially expressed proteins based on quantitative proteomics. (**D)** Gene Ontology enrichment analysis for differentially expressed proteins. Apoptotic signaling pathways are highlighted in red. (**E)** Heatmap for expression of apoptosis-related proteins in *DDRGK1* knockout cells and the control cells. (**F-G)** *DDRGK1* knockout A549 (F) and H1299 (G) cells were treated with cisplatin (20 μM, 48 h) and cell were harvested for Annexin V-FITC/PI staining and flow cytometry analysis. These are related to Figure 7D. (**H-I)** Plate colony formation detecting clonogenic ability of *DDRGK1* knockout A549 (H) and H1299 (I) cells under cisplatin treatment as described in method. These are related to Figure 7F.

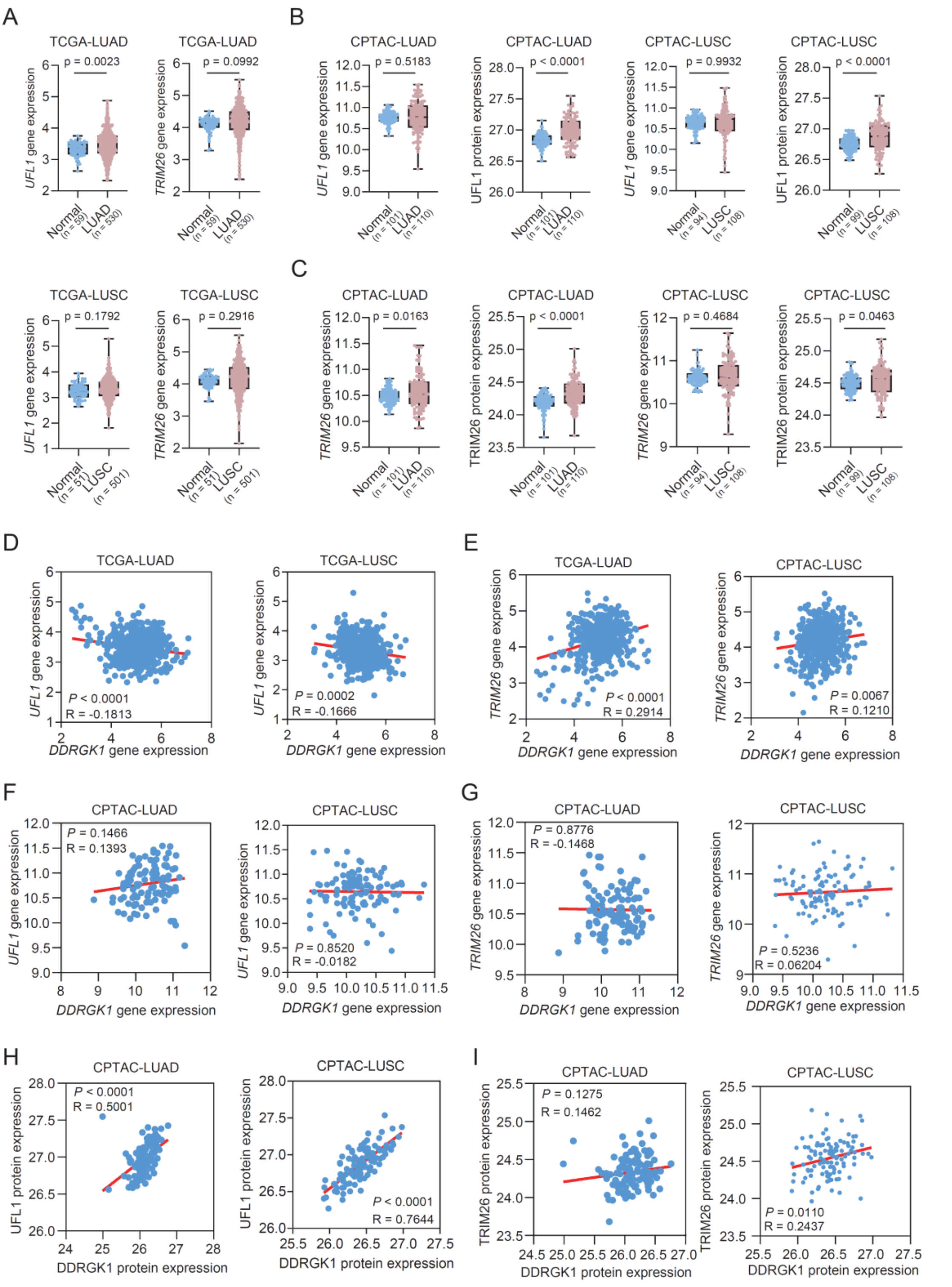

Figure S7 The analysis of TRIM26 and UFL1 expression together with their correlation to DDRGK1 in LUAD and LUSC using TCGA and CPTAC datasets. (A) Analysis of *UFL1* and *TRIM26* mRNA expression levels in LUAD/LUSC tissues and normal lung tissues from TCGA database. **(B)** Analysis of UFL1 mRNA and protein expression levels in LUAD/LUSC tissues and normal lung tissues from CPTAC

database. **(C)** Analysis of TRIM26 mRNA and protein expression levels in LUAD/LUSC tissues and normal lung tissues from CPTAC database. **(D-E)** Pearson correlation analysis was performed to assess the correlations between mRNA expression levels of *DDRGK1* and *UFL1* **(D)**, as well as between *DDRGK1* and *TRIM26* **(E)** in TCGA-LUAD and TCGA-LUSC. **(F-G)** Pearson correlation analysis was performed to assess the correlations between mRNA expression levels of *DDRGK1* and *UFL1* **(F)**, as well as between *DDRGK1* and *TRIM26* **(G)** in CPTAC-LUAD and CPTAC-LUSC. **(H-I)** Pearson correlation analysis was performed to assess the correlations between protein expression levels of DDRGK1 and UFL1 **(H)**, as well as between DDRGK1 and TRIM26 **(I)** in CPTAC-LUAD and CPTAC-LUSC.
